# Accurately Controlled Tumor Temperature with Silica-Coated Gold Nanorods for Optimal Immune Checkpoint Blockade Therapy

**DOI:** 10.34133/bmr.0024

**Published:** 2024-04-29

**Authors:** Wan Su Yun, Wonseok Yang, Man Kyu Shim, Sukyung Song, Jiwoong Choi, Jeongrae Kim, Jinseong Kim, Yujeong Moon, SeongHoon Jo, Dong-Kwon Lim, Kwangmeyung Kim

**Affiliations:** ^1^ College of Pharmacy, Graduate School of Pharmaceutical Sciences, Ewha Womans University, Seoul 03760, Republic of Korea.; ^2^ KU-KIST Graduate School of Converging Science and Technology, Korea University, 145 Anam-ro, Seongbuk-gu, Seoul 02841, Republic of Korea.; ^3^ Medicinal Materials Research Center, Biomedical Research Division, Korea Institute of Science and Technology (KIST), Seoul02792, Republic of Korea.; ^4^ Department of Integrative Energy Engineering, Korea University, 145 Anam-ro, Seongbuk-gu, Seoul 02841, Republic of Korea.; ^5^ Brain Science Institute, Korea Institute of Science and Technology (KIST), 5, Hwarang-ro 14-gil, Seongbuk-gu, Seoul 02792, Republic of Korea.

## Abstract

Photothermal therapy (PTT) at mild temperatures ranging from 44 to 45 °C holds tremendous promise as a strategy for inducing potent immunogenic cell death (ICD) within tumor tissues, which can reverse the immunosuppressive tumor microenvironment (ITM) into an immune-responsive milieu. However, accurately and precisely controlling the tumor temperature remains a formidable challenge. Here, we report the precision photothermal immunotherapy by using silica-coated gold nanorods (AuNR@SiO_2_), and investigating the optimal administration routes and treatment protocols, which enabled to achieve the sustained and controlled mild heating within the tumor tissues. First, the highest photothermal performance of AuNR@SiO_2_ with 20-nm silica shell thickness than 5 or 40 nm was confirmed in vitro and in vivo. Then, the optimal conditions for precision immunotherapy were further investigated to produce mild temperature (44 to 45 °C) accurately in tumor tissues. The optimal conditions with AuNR@SiO_2_ result in a distinct cell death with high early/late apoptosis and low necrosis, leading to very efficient ICD compared to lower or higher temperatures. In colon tumor-bearing mice, intratumorally injected AuNR@SiO_2_ efficiently promotes a mild temperature within the tumor tissues by local irradiation of near-infrared (NIR) laser. This mild PTT substantially increases the population of mature dendritic cells (DCs) and cytotoxic T cells (CTLs) within tumor tissues, ultimately reversing the ITM into an immune-responsive milieu. Furthermore, we found that the combination mild PTT with AuNR@SiO_2_ and anti-PD-L1 therapy could lead to the 100% complete regression of primary tumors and immunological memory to prevent tumor recurrence. Collectively, this study demonstrates that AuNR@SiO_2_ with a robust methodology capable of continuously inducing mild temperature accurately within the ITM holds promise as an approach to achieve the precision photothermal immunotherapy.

## Introduction

Photothermal therapy (PTT) can efficiently induce immunogenic cell death (ICD) in tumor cells, which is increasingly recognized as a potent approach for the precise cancer immunotherapy owing to its highly localized effectiveness with ability to meticulously control the temperature within the tumor microenvironment in a noninvasive manner [1,2]. The ICD in the tumor cells during PTT leads to the translocation of intracellular calreticulin (CRT) to the plasma membrane surface, accompanied by the extracellular release of heat shock protein 70 (HSP70), high-mobility group box 1 (HMGB1), and adenosine triphosphate (ATP) [3]. These molecular signals, emitted from tumor cells undergoing ICD, are referred to as damage-associated molecular patterns (DAMPs), which play a crucial role in facilitating interactions with the host’s immune system to promote “find and eat me” signals [4]. This sequential interplay results in dendritic cell (DC) maturation and T cell activation, leading to an increase in tumor-infiltrating lymphocytes (TILs) within the tumor microenvironment [5]. Consequently, it efficiently reverses the immunosuppressive tumor microenvironment (ITM) into an immune-responsive milieu that is favorable to anticipating remarkable responses to cancer immunotherapy, particularly immune checkpoint blockade (ICB) therapies [6,7].

To utilize the PTT effect for cancer immunotherapy, nanomaterials with various sizes and shapes of organic polymers, semiconducting polymers, carbon materials, and metallic structures have been extensively investigated to deliver an external light dose into the tumor for local heating [8–14]. Traditionally, achieving an efficient tumor ablation requires localized heating to temperatures exceeding 50 °C, resulting in a harsh tumor microenvironment that undergoes irreversible damage on both tumor and healthy tissues [15]. Notably, inflammatory responses from the tumor cells following PTT at excessively high temperatures can lead to the release of immunosuppressive cytokines, which hinder the antitumor immune responses. In contrast, very low temperature was not sufficient to elicit a robust antitumor immune response and to eradicate the tumor tissues and the remaining tumor margin via the abscopal effect [16,17]. Recently, PTT at relatively mild temperature (mild PTT) in the range of 44 to 45 °C is being typically applied in tumor treatment for effectively inducing ICD within the tumor cells to promote substantial DAMPs and thereby improve the immunogenicity within the ITM [18]. In this regard, AuNRs have been attractive materials for mild PTT because of the simple structure, robust synthetic protocol, and a highly efficient photothermal effect with tunable absorption wavelengths in the near-infrared (NIR) ranges [19]. However, the photostability, biosafety, and photothermal performance of AuNRs should be further improved because of particle aggregation in physiological conditions and possible cytotoxicity of surfactant on AuNRs [20]. While bare AuNRs show the poor photothermal conversion efficiency due to their unstable nature that hinders the collection of sufficient energy to induce tumor cell death [21], it was well known that the formation of silica shell on AuNRs could improve the PTT performance, structural photostability, and biocompatibility [22]. We previously reported a robust silica shell formation method in nanoscale on AuNRs, which can accurately control the silica shell thickness from 5 to 40 nm [23]. The silica shell with 20 nm on AuNRs showed the highest photothermal (PT) effect in test tube, but the performance of silica shell-coated AuNRs (AuNR@SiO_2_) has not been proven yet in tissue and has not proven the performance for cancer immunotherapy.

Precise control of temperature within the tumor microenvironment through PTT has been challenging in cancer immunotherapy. Furthermore, the clinical translation of gold-based nanomaterials for PTT has been impeded due to the toxicity associated with high doses and limited biodegradability after intravenous (I.V.) administration [24]. To address these problems, there are valid rationale and a strong need to absolutely reduce the dosage of nanomaterials via optimization of administration route capable of precise control of temperature in tumor tissues [25]. This has the great potential to trigger an outstanding antitumor immune response in the tumor microenvironment via mild PTT, achieving complete primary tumor regression and also preventing the growth of metastatic tumors by the abscopal effect while avoiding side effects within the body according to reduced material dosage [26]. Therefore, extensive optimization studies involving new design of materials, administration routes, and detailed treatment protocols for achieving sustained and controlled mild heating within the tumor tissues can provide a valuable strategy in cancer immunotherapy.

**Fig. 1.**
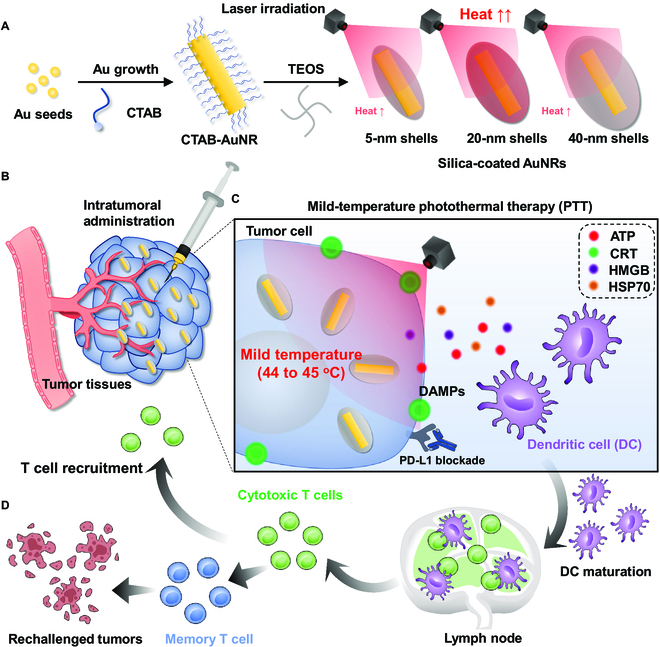
The schematic illustration showing the strategy for mild-temperature photothermal therapy (PTT) using silica-coated gold nanorods (AuNR@SiO_2_) to potentiate immune checkpoint blockade therapy. (A) AuNR@SiO_2_ with silica shell thickness of 5, 20, or 40 nm (AuNR@SiO_2_-5, AuNR@SiO_2_-20, and AuNR@SiO_2_-40, respectively) was prepared, and their photothermal efficiency was compared both in vitro and in vivo. (B) In colon tumor-bearing mice, I.T. injected AuNR@SiO_2_-20, which demonstrates superior photothermal efficiency compared to AuNR@SiO_2_-5 and AuNR@SiO_2_-40, induced a mild temperature (45 °C) precisely and reproducibly within the tumor tissues locally irradiated with laser irradiation, resulting in potent immunogenic cell death (ICD) accompanying high DAMPs, such as calreticulin (CRT), high-mobility group box 1 (HMGB1), and adenosine triphosphate (ATP). (C) Mild PTT with AuNR@SiO_2_-20 significantly increased tumor-infiltrating mature dendritic cells (DCs) and cytotoxic T cells (CTLs) within the tumor tissues, thereby reversing the ITM into immune-responsive milieu and ultimately inducing potent antitumor immune responses. (D) The combination of AuNR@SiO_2_-20-mediated mild PTT with anti-PD-L1 antibody led to a complete regression of the primary tumors and also effectively establish the immunological memory to prevent the recurrence according to tumor rechallenge.

In this study, we propose experimental model to demonstrate the performance of silica shell-coated AuNRs for producing accurately controlled local temperature in the ranges of 44 to 45 °C within tumor tissues. For the optimal mild PTT in cancer immunotherapy, the photothermal efficiency of size-configurable AuNR@SiO_2_ with silica shell thickness of 5, 20, or 40 nm (AuNR@SiO_2_-5, AuNR@SiO_2_-20, and AuNR@SiO_2_-40, respectively) is compared to determine the optimal gold material for inducing a mild temperature of 44 to 45 °C within tumor tissues (Fig. 1A). We postulated that optimal AuNR@SiO_2_ can induce a distinct cell death mechanism with high early/late apoptosis by promoting a mild temperature within the tumor cells, resulting in potent ICD accompanying high DAMPs compared to lower or higher temperatures following laser irradiation. In colon tumor-bearing mice, intratumorally (I.T.) injected AuNR@SiO_2_ precisely and reproducibly induces a mild PTT with 44.5 °C within the tumor tissues locally irradiated with laser irradiation (Fig. 1B). Hence, mild PTT with AuNR@SiO_2_ leads to a significant increase in tumor-infiltrating mature DCs and cytotoxic T cells (CTLs) within the tumor tissues, thereby reversing the ITM into immune-responsive milieu and ultimately eliciting robust antitumor immune responses (Fig. 1C). Finally, the combinatorial immunotherapy of AuNR@SiO_2_-mediated mild PTT with anti-PD-L1 antibody results in a complete regression of the primary tumors and also effectively prevents the recurrence according to tumor rechallenge by establishing the immunological memory during treatments (Fig. 1D).

## Materials and Methods

### Reagents

Gold (III) chloride trihydrate, silver nitrate, hydrochloric acid, tetraethyl orthosilicate (TEOS), (3-aminopropyl)trimethoxysilane (APTMS), l-ascorbic acid, Annexin V/PI staining kit, and sodium borohydride were purchased from Sigma-Aldrich (Oakville, ON, USA). Cetyltrimethylammonium bromide (CTAB) was purchased from Tokyo Chemical Industry (TCI; Tokyo, Japan). Cyanine5.5 NHS ester was purchased from Lumiprobe (Hunt Valley, MD, USA). Transmission electron microscopy (TEM) grid (Carbon Film 200 Mesh copper) was purchased from Electron Microscopy Sciences (Hatfield, PA, USA). RPMI 1640 medium, fetal bovine serum (FBS), penicillin, and streptomycin were purchased from WELGENE Inc. (Daegu, Korea). FBS and Dulbecco’s phosphate-buffered saline (DPBS) were purchased from Thermo Fisher Scientific (Waltham, MA, USA). Cell counting kit-8 (CCK-8) was purchased from Vitascientific (Beltsville, MD, USA). The terminal deoxynucleotidyl transferase–mediated deoxyuridine triphosphate nick end labeling (TUNEL) assay kit was purchased from R&D Systems (Minneapolis, MN, USA). Anti-mouse HMGB1 antibody (ab18256), anti-mouse HSP70 (ab2787), and anti-mouse CRT (ab196159) antibody were purchased from Abcam (Hanam, Republic of Korea). Fluorescent dye-conjugated antibodies against mouse CD45.2 (catalog no. 109806), mouse CD44 (catalog no. 103024), mouse CD8a (catalog no. 100724), mouse CD40 (catalog no. 124609), mouse CD62L (catalog no. 104406), mouse CD11c (catalog no. 117309), mouse CD3 (catalog no. 100219), and mouse CD86 (catalog no. 105005) were purchased from BioLegend (San Diego, CA, USA). CT26 (mouse colon adenocarcinoma) was purchased from the American Type Culture Collection (ATCC; Manassas, VA, USA).

### Preparation of CTAB-AuNRs

AuNRs were synthesized by the seed-mediated growth method with CTAB [27]. First, the seed solution was prepared by mixing 9.75 ml of CTAB (0.1 M) with 0.25 ml of HAuCl_4_ (0.01 M) and 0.6 ml of a freshly prepared ice-cold NaBH_4_ solution (0.01 M). The solution was stirred vigorously for 1 to 2 min and then maintained at 28 °C for 3 h. In the second step, a growth solution was prepared by adding 25 ml of HAuCl_4_ (0.01 M), 4.0 ml of AgNO_3_ (0.01 M), 4.0 ml of freshly prepared ascorbic acid (0.01 M), and 5.0 ml of HCl (1.0 M) to 450 ml of CTAB (0.1 M). A seed solution (5.0 ml) was added to this solution, and the reaction mixture was subjected to quick shaking for 2 s and then kept undisturbed overnight at 28 °C. The solution color changed to purple with the formation of the AuNRs.

### Preparation of AuNR@SiO_2_

First, the excess CTAB in the prepared AuNR solution should be removed. AuNR solution (10 ml) was centrifuged twice at 15,000 rcf and 10,000 rcf for 15 min. After discarding the supernatant, the AuNRs were redispersed in distilled water (DW) to set the optical density (OD) at ~1.0. The AuNR solution (9.0 ml; OD at 780 nm: 1.0) was transferred to a 50-ml falcon tube. Isopropanol (IPA, 13.5 ml) was added, and the solution was gently stirred, followed by the addition of 1.0 ml of 0.05 M NaOH. Finally, a variable amount (1,100, 1,250, and 1,400 μl) of TEOS solution (0.05 M in IPA) was added under slow shaking [23]. The solution mixture was gently shaken for 24 h. The solution was then centrifuged at 10,000 rcf for 15 min, the supernatant was discarded, and the precipitates were redispersed in DW (9.0 ml).

### Characterization of AuNR@SiO_2_

The morphology of AuNR@SiO_2_ in DW was observed via TEM (Talos F200X; FEI Company, USA). The hydrodynamic size and zeta potential of AuNR@SiO_2_ in DW (200 μg/ml) were measured via dynamic light scattering (DLS) instrument (Zetasizer Nano ZS, Malvern Instruments, Malvern, UK). The stability of AuNR@SiO_2_ in DW at 37 °C was evaluated using DLS for 7 days. To investigate the photothermal conversion efficiency and the photothermal stability of CTAB-AuNR, and various thicknesses of AuNR@SiO_2_, 1 ml of solution (200 μg/ml) in an eppendorf tube (EP) tube was irradiated with an 808-nm NIR laser at 1 W cm^−2^ for 10 min and then cooled naturally to room temperature for 12 min without laser irradiation by repeating four cycles. The temperature changes were monitored every 10 s.

### In vitro cytotoxicity and cellular uptake of AuNR@SiO_2_

CT26 (murine colorectal carcinoma cell) was cultured in a medium containing 10% FBS and 1% antibiotics at 37 °C in a CO_2_ incubator. The cytotoxicity assay of AuNR@SiO_2_ was carried out using CCK-8. CT26 cells (1 × 10^4^) were seeded in 96-well cell culture plates and stabilized for 24 h. CT26 cells were treated with various concentrations (ranging from 0 to 1,600 μg/ml) of 5-, 20-, and 40-nm AuNR@SiO_2_ at 37 °C for 24 h, followed by incubation with a culture medium containing 10% CCK-8 solution for 30 min. The viabilities of all subject cells were calculated by a microplate reader with 450 nm of wavelength. The concentration-dependent cell viabilities were plotted and fitted with GraphPad Prism 8 software (GraphPad Software, San Diego, CA, USA). To investigate the cellular uptake behavior of AuNR@SiO_2_, 1 × 10^5^ of CT26 cells were seeded in a glass-bottom confocal dish. After 24 h of stabilization, CT26 was treated with various thicknesses of AuNR@SiO_2_ (5, 20, and 40 nm) at 37 °C for 24 h, subsequently washed with PBS, fixed with 4% paraformaldehyde for 15 min, and stained with 4′,6-diamidino-2-phenylindole (DAPI) for 15 min. Fluorescence images of AuNR@SiO_2_-treated cells were obtained with a confocal laser scanning microscope (CLSM; Leica TCS SP8, Leica Microsystems GmbH, Wetzlar, Germany) equipped with diode (405 nm), Ar (458, 488, 514 nm), and He-Ne (633 nm) lasers. Dark-field images of AuNR@SiO_2_-treated cells were obtained with a upright fluorescence microscope (Zeiss Axio Imager M1, Carl Zeiss, Oberkochen, Germany) and quantified using Image-Pro software (Media Cybernetics, Rockville, MD, USA) to identify the intracellular distribution of AuNR@SiO_2_.

### In vitro antitumor activity of AuNR@SiO_2_ under laser irradiation

For in vitro antitumor activity of AuNR@SiO_2_ under laser irradiation, 1 × 10^4^ CT26 cells were seeded in 96-well cell culture plate and stabilized for 24 h. CT26 cells were treated with 50, 100, and 200 μg/ml of 20-nm AuNR@SiO_2_ at 37 °C for 24 h, followed by 808-nm NIR laser irradiation at 1 W cm^−2^ for 4 min. After 24-h incubation in dark condition, the RPMI medium was removed and the plate was washed with PBS twice. The cells were incubated with the medium containing 10% CCK-8 solution for 30 min, and the absorbance (450 nm) was measured with a microplate reader (VERSAmax; Molecular Devices Corp., USA).

### In vitro apoptosis study and DAMP analysis

CT26 cells were seeded in 100-pi cell culture dishes at a density of 2 × 10^6^ and stabilized for 24 h. CT26 cells were treated with 50, 100, and 200 μg/ml of 20-nm AuNR@SiO2 at 37 °C for 24 h, followed by 808-nm NIR laser irradiation at 1 W cm^−2^ for 4 min. The cells were collected and stained by Annexin V/PI Cell Apoptosis Kit following the manufacturer’s protocol. The cells were finally analyzed with a flow cytometer (BD FACSVerse, BD Biosciences, USA). The surface-exposed CRT expression and extracellular release of HMGB1, ATP, and HSP70 were analyzed to examine the DAMPs from CT26 under laser irradiation. First, 2 × 10^6^ CT26 cells were seeded in 100-pi cell culture dishes, followed by incubation with AuNR@SiO_2_ (50, 100, and 200 μg/ml) at 37 °C for 24 h. Then, cells were irradiated with 808-nm NIR laser irradiation at 1 W cm^−2^ for 4 min. After 24 h, cells were stained with CRT antibody at 4 °C for 12 h, followed by analysis using a CLSM. Second, supernatants were collected to analyze the HMGB1 and HSP70 released from the cells via Western blot and to analyze the released ATP using a commercial ATP assay kit.

### In vivo distribution of AuNR@SiO_2_ in colon tumor-bearing mice

Mice were bred under pathogen-free conditions at the Ewha Womans University. All experiments with live animals were performed in compliance with the relevant laws and institutional guidelines of the Institutional Animal Care and Use Committee (IACUC) in Ewha Womans University, and the IACUC approved the experiment (approved number EWHA IACUC 22-073-2). Murine colon tumor models were prepared by subcutaneous inoculation of 1 × 10^6^ CT26 cells into the flanks of the mice. After that, CT26 tumor-bearing mice were I.T. injected with AuNR@SiO_2_ (0.1 mg/kg) and I.V. injected with AuNR@SiO_2_ (5, 10, or 25 mg/kg) when tumor volumes grew to approximately 100 mm^3^. NIRF imaging of mice was performed via an IVIS Lumina Series III system (PerkinElmer, Waltham, MA, USA).

### In vivo tumor inhibition and immune response

CT26 tumor-bearing mice were randomly divided into five groups: (a) control, (b) laser, (c) 50 μg/kg (+L), (d) 100 μg/kg (+L), and (e) 200 μg/kg (+L). Then, mice were treated on day 0 with AuNR@SiO_2_ (50, 100, or 200 μg/kg) with I.T. injection, at which time tumor volumes had reached 50 to 80 mm^3^. Tumor tissues were irradiated with a 1 W cm^−1^ laser (MDL-III-808-2W, Changchun New Industries Optoelectronics Tech. Co. Ltd., Jilin, China) for 4 min under dark condition. Therapeutic efficacy was assessed by measuring tumor volumes, calculated as (the largest diameter) × (the smallest diameter)^2^ × 0.53, every 2 days, wherein the mice with measurements exceeding tumor volumes of 2,000 mm^3^ were considered dead. For the analysis of tumor-infiltrating immune cells, tumor tissues were collected on day 7, and single cells were isolated from tissues using a tumor dissociation kit (Miltenyi Biotec). Following cell counting, the single cells were incubated with FcBlock for 5 min to avoid nonspecific binding. Then, multiparameter staining was performed for 1 h at 4 °C to identify the following populations in tumor tissues: (a) CD8^+^ T cells (CD45^+^CD3^+^CD8^+^), (b) matured DCs (CD11c^+^CD40^+^CD86^+^), and (c) T_regs_ (CD45^+^CD3^+^CD4^+^CD25^+^) [17]. To assess the enhanced therapeutic efficacy and antitumor immunity by combinatory PTT and ICB, anti-PD-L1 antibody (10 mg/kg) was simultaneously administered via intraperitoneal (I.P.) injection [28]. On day 50 after treatment, the systemic immunological memory in CR mice that experience CR in 100 and 200 μg/kg groups was evaluated by tumor rechallenge with CT26 cells (1 × 10^6^) at the opposite flank. Rechallenged tumor volumes were measured every 2 days, and splenic effector/memory CD8^+^ T cells, defined as CD3^+^CD8^+^CD44^+^CD62L^low^, were analyzed by flow cytometry. Finally, IFN-γ, TNF-α, and IL-6 in the serum were analyzed using an enzyme-linked immunosorbent assay (ELISA) kit at the endpoint (day 79 after first tumor inoculation).

### Statistical analysis

Statistical significance between two groups was analyzed using Student’s *t* test. In case of more than two groups, one-way analysis of variance (ANOVA) was used, and multiple comparisons were performed using Tukey–Kramer post hoc test. Survival results were plotted using Kaplan−Meier curves and analyzed using the log-rank test. All results are presented as mean ± SD, and *P* values of <0.05*, <0.01**, and <0.001*** were considered statistically significant.

## Results and Discussion

### Physicochemical properties of silica-coated gold nanorods

**Fig. 2.**
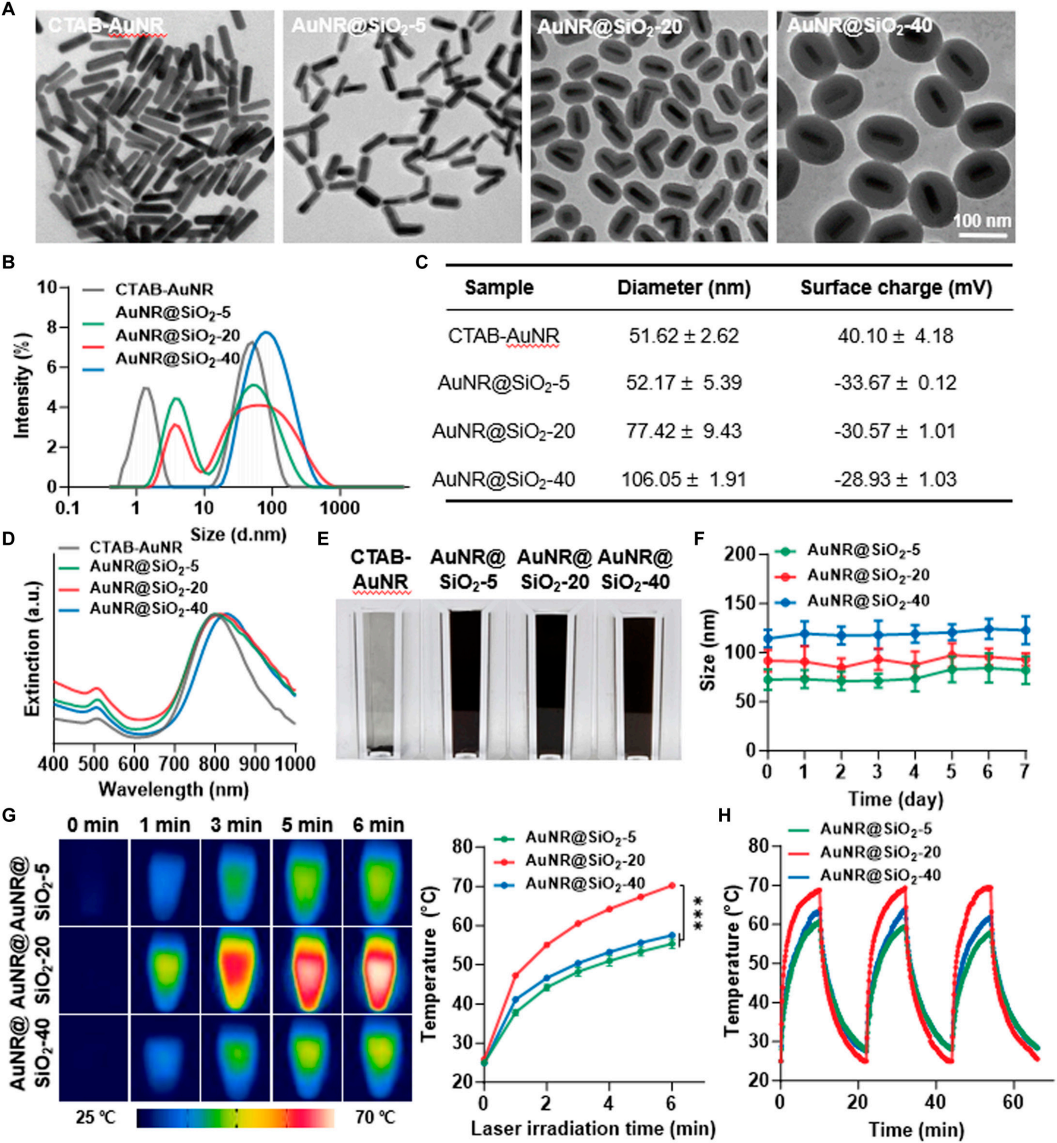
Comparative studies on the characteristics of AuNR@SiO_2_-5, AuNR@SiO_2_-20, and AuNR@SiO_2_-40. (A) TEM images of CTAB-AuNR, AuNR@SiO_2_-5, AuNR@SiO_2_-20, and AuNR@SiO_2_-40 in DW at a concentration of 1 mg/ml. (B and C) Average size and surface charge of CTAB-AuNR, AuNR@SiO_2_-5, AuNR@SiO_2_-20, and AuNR@SiO_2_-40 in saline at a concentration of 1 mg/ml. (D) UV–Vis spectra of CTAB-AuNR, AuNR@SiO_2_-5, AuNR@SiO_2_-20, and AuNR@SiO_2_-40. (E) Optical images of CTAB-AuNR, AuNR@SiO_2_-5, AuNR@SiO_2_-20, and AuNR@SiO_2_-40 after 24 h of dispersion in saline. (F) Size stability of CTAB-AuNR, AuNR@SiO_2_-5, AuNR@SiO_2_-20, and AuNR@SiO_2_-40 in saline for 7 days. (G) Photothermal images of CTAB-AuNR, AuNR@SiO_2_-5, AuNR@SiO_2_-20, and AuNR@SiO_2_-40 in tubes following laser irradiation for varying durations with a power of 1.0 W/cm^2^. (H) Multi-cycle irradiation study for CTAB-AuNR, AuNR@SiO_2_-5, AuNR@SiO_2_-20, and AuNR@SiO_2_-40.

A significant enhancement in photothermal effects and photostability could be expected via silica-coated gold nanorods (AuNR@SiO_2_), which is mainly attributed to the lower interfacial thermal resistance owing to a silica-coated surface on AuNRs [29]. For the comparative studies to determine the effective photothermal gold materials for mild PTT, AuNR@SiO_2_ with varying silica shell thickness of 5, 20, or 40 nm was prepared via previously reported base and solvent conditions [23]. Briefly, CTAB-AuNRs were synthesized through a seed-mediated growth method, followed by reactions with varying quantities of TEOS (1,100, 1,250, and 1,400 μl for target silica shell thickness of 5, 20, and 40 nm, respectively; Fig. S1). TEM images clearly showed that each AuNR@SiO_2_ possessed a silica-coated surface, with the exact target silica shell thickness increasing gradually in accordance with the amount of TEOS, resulting in AuNR@SiO_2_ with 5-nm (AuNR@SiO_2_-5), 20-nm (AuNR@SiO_2_-20), or 40-nm (AuNR@SiO_2_-40) silica shells (Fig. 2A). In addition, the average diameter in aqueous conditions increased as the surface silica-coated areas thickened from CTAB-AuNRs (51.62 ± 2.62 nm) to AuNR@SiO_2_-5 (52.17 ± 5.39 nm), AuNR@SiO_2_-20 (77.42 ± 9.43 nm), and AuNR@SiO_2_-40 (106.05 ± 1.91 nm; Fig. 2B and C) [30]. The zeta potential of CTAB-AuNRs shifted to a negative charge after the surface coating with silica shells, but there were no significant differences in the surface charges of AuNR@SiO_2_-5, AuNR@SiO_2_-20, and AuNR@SiO_2_-40. Furthermore, ultraviolet–visible (UV–Vis) spectral shape of CTAB-AuNRs was not significantly changed after silica shell coating, but slight red-shifts in the peak plasmon band of AuNR@SiO_2_ were observed due to the higher local refractive index of the silica shells (1.45) compared to water (1.33; Fig. 2D). Importantly, colloidal stability of CTAB-AuNR was greatly improved by surface silica-coating, wherein all AuNR@SiO_2_ was well dispersed in aqueous conditions for 24 h compared to CTAB-AuNR showing obvious precipitation (Fig. 2E). Furthermore, AuNR@SiO_2_ showed a remarkably stable characteristics in saline with no significant changes in average size for 7 days (Fig. 2F and Fig. S2). The photothermal efficiency of AuNR@SiO_2_-5, AuNR@SiO_2_-20, and AuNR@SiO_2_-40 was compared via tube assays under laser irradiation with power of 1.0 W/cm^2^. Photothermal images demonstrated a substantial increase in local temperature within the tubes by AuNR@SiO_2_-20 compared to AuNR@SiO_2_-5 and AuNR@SiO_2_-40 upon laser irradiation (Fig. 2G). Quantitatively, AuNR@SiO_2_-20 elevated the local temperature to 70.23 ± 0.6 °C within the tubes after 6 min of laser irradiation, which is significantly higher than AuNR@SiO_2_-5 (55.37 ± 1.17 °C) and AuNR@SiO_2_-40 (57.53 ± 0.48 °C). This is because a silica shell on AuNR surface thicker than 20 nm can lead to a decrease in the local fluence of incident laser, attributable to increased light scattering [30]. Finally, photothermal conversion efficiency and stability of AuNR@SiO_2_-5, AuNR@SiO_2_-20, and AuNR@SiO_2_-40 were assessed through a multi-cycle irradiation study. All AuNR@SiO_2_ with different silica shell thickness experienced no loss of photothermal conversion efficiency that is observed by a decrease in photothermal effects during successive cycles of laser irradiation compared to the initial irradiation, across three on/off cycles (Fig. 2H). However, the maximal temperature in the tubes after laser irradiation remained consistently higher in AuNR@SiO_2_-20 group throughout the multi-cycle irradiation compared to AuNR@SiO_2_-5 and AuNR@SiO_2_-40. These characterization results indicate that AuNR@SiO_2_-20 is highly suitable of promoting local hyperthermia under laser irradiation owing to its outstanding photothermal efficiency compared to AuNR@SiO_2_-5 and AuNR@SiO_2_-40. To promote the mild temperature effectively within tumor tissues via a low material dosage, AuNR@SiO_2_-20 that exhibits an outstanding photothermal efficiency was selected as a photothermal agent in this study.

### In vitro studies to promote potent ICD by inducing mild PTT in the tumor cells

**Fig. 3.**
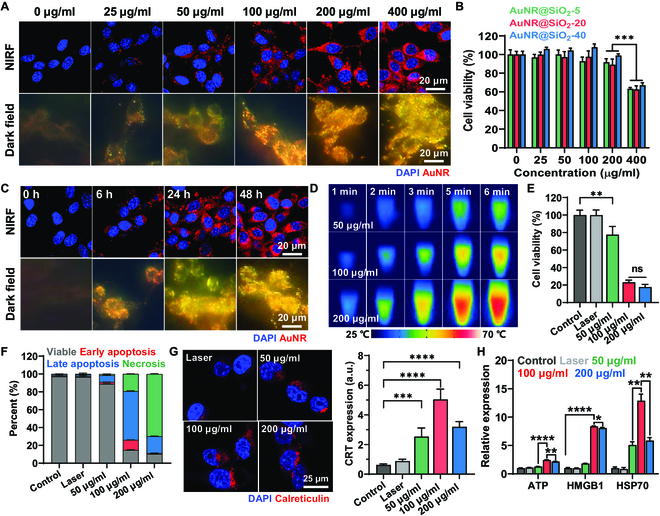
In vitro optimization studies to promote potent ICD by inducing mild PTT in the tumor cells. (A) NIRF and dark field images of CT26 cells treated with varying concentrations (0 to 400 μg/ml) of AuNR@SiO_2_-20 for 48 h. (B) CT26 cell viability after 48 h of treatment with AuNR@SiO_2_-20 at concentration ranging from 0 to 400 μg/ml. (C) NIRF and dark field images of CT26 cells treated with 200 μg/ml AuNR@SiO_2_-20 for 0, 3, 6, 24, or 48 h. (D) Photothermal images of CT26 cells treated with 50, 100, or 200 μg/ml upon laser irradiation. (E) CT26 cell viability after 24 h of treatment with 50, 100, or 200 μg/ml AuNR@SiO_2_-20 upon laser irradiation for 3 min with power of 1.0 W/cm^2^. (F) Quantification of percentages of live (Annexin^−^PI^−^), early apoptosis (Annexin^+^PI^−^), late apoptosis (Annexin^+^PI^+^), and necrosis (Annexin^−^PI^+^) in CT26 cells after 24 h of treatment with 50, 100, or 200 μg/ml AuNR@SiO_2_-20 upon laser irradiation for 3 min with power of 1.0 W/cm^2^. (G and H) Expression of (G) CRT and (H) extracellular release of ATP, HMGB1, and HSP70 from CT26 cells after 24 h of treatment with 50, 100, or 200 μg/ml AuNR@SiO_2_-20 upon laser irradiation for 3 min with power of 1.0 W/cm^2^.

The optimal conditions for inducing a mild temperature of 44 to 45 °C via AuNR@SiO_2_-20 in cell cultured system were investigated in cultured CT26 colon carcinoma cells. First, CT26 cells were treated with different concentrations of 1 wt % NIR fluorescence (NIRF) dye, Cy5.5, labeled AuNR@SiO_2_-20 (Cy5.5-AuNR@SiO_2_-20) ranging from 0 to 400 μg/ml for 48 h, wherein a robust cellular uptake was clearly observed in both NIRF and dark field images (Fig. 3A). The cellular uptake of Cy5.5-AuNR@SiO_2_-20 (red color) within the tumor cells increased gradually as the treatment concentration was increased from 0 to 200 μg/ml, but that was similar in cells treated with 200 or 400 μg/ml, indicating that the maximum cellular uptake of AuNR@SiO_2_-20 is observed at the concentration of 200 μg/ml (Fig. S3A). Notably, 48-h treatment with 400 μg/ml of Cy5.5-labeled AuNR@SiO_2_-20 induced a significant cytotoxicity, leading to a notable decrease in CT26 cell viability compared to cells treated with 0 to 200 μg/ml (Fig. 3B). The nonspecific toxicity resulting from the high concentration of Cy5.5-AuNR@SiO_2_-20 in the absence of laser irradiation may induce severe cytotoxicity toward normal cells as well as tumor cells. In addition, cellular uptake of Cy5.5-AuNR@SiO_2_-20 gradually increased in an incubation time-dependent manner after treatment with 200 μg/ml. However, there were no significant differences in the amount of cellular uptake of Cy5.5-AuNR@SiO_2_-20 in CT26 cells after 24 or 48 h of treatment (Fig. 3C and Fig. S3B). Therefore, these findings suggest that 24 h of treatment with 200 μg/ml Cy5.5-AuNR@SiO_2_-20 is an optimal concentration that can expect a maximum cellular uptake without significant cytotoxicity.

To evaluate the levels of ICD within tumor cells according to a different temperature induced by AuNR@SiO_2_-20 upon laser irradiation, CT26 tumor cells were treated with 50, 100, or 200 μg/ml of AuNR@SiO_2_-20 for 24 h, followed by analysis of temperature after laser irradiation with power of 1.0 W/cm^2^. The photothermal images showed that tumor cells exhibited a low (41 °C), mild (45 °C), and high (50.77 °C) temperatures after treatment with 50, 100, or 200 μg/ml of AuNR@SiO_2_-20 for 24 h and subsequent laser irradiation with 3 min, respectively (Fig. 3D and Fig. S4). These results demonstrate that the temperature within the tumor cells in cultured system can be accurately controlled using AuNR@SiO_2_-20. As expected, the viability of CT26 cells significantly decreased along with increase in the AuNR@SiO_2_-20 concentration from 50 to 100 μg/ml upon laser irradiation, while that was similar between cells treated with 100 or 200 μg/ml (Fig. 3E). These results indicate that treatment with 50 μg/ml of AuNR@SiO_2_-20 resulted in a very low temperature (41 °C) to induce sufficient cell death within the tumor cells, with only about 30% of cell death observed. As a control, there was no significant decrease in CT26 cell viability after exposure to laser irradiation alone. Furthermore, the levels of cell death were nearly similar, with approximately 20% cell viability when CT26 cells were exposed to a mild temperature of 45 °C or a higher temperature of 50 °C induced by 100 or 200 μg/ml of AuNR@SiO_2_-20 treatment, respectively. Importantly, the patterns of tumor cell death, as evaluated by Annexin V/propidium iodide (PI) staining, were clearly different after treatment with 100 or 200 μg/ml of AuNR@SiO_2_-20 for 24 h, followed by laser irradiation at a power of 1.0 W/cm^2^ for 3 min (Fig. 3F). When the CT26 cells were treated with 100 μg/ml of AuNR@SiO_2_-20 and laser irradiation, high levels of early/late apoptosis (Annexin V^+^PI^−^; 11.38 ± 0.11%/Annexin V^+^PI^+^; 54.57 ± 0.51%) with low necrosis (Annexin V^−^PI^+^; 19.28 ± 0.25%) were predominantly observed, whereas the rate of necrosis (69.82 ± 0.57%) was significantly increased along with low levels of early/late apoptosis (0.91 ± 0.16%/18.83 ± 0.29%) in cells treated with 200 μg/ml of AuNR@SiO_2_-20 and laser irradiation. These distinct cell death mechanisms promoted by AuNR@SiO_2_-20-mediated mild PTT can lead to effective ICD, thereby eliciting enhanced antitumor immune responses. This is because the tumor cells in the early apoptosis state expose “find-me and eat-me” signals through surface CRT and phosphatidylserine expressions; in addition, late apoptotic tumor cells release soluble DAMPs, such as HMGB1, ATP, and HSP, resulting in maturation and phagocytosis of DCs to up-regulate the engulfment machinery [31,32]. In contrast, necrosis of tumor cells is usually considered immunologically harmful due to active exposure to the “do not eat-me” signal through surface CD47 expression [33]. As a control, CT26 cells treated with 50 μg/ml of AuNR@SiO_2_-20 and laser irradiation exhibited only a small quantity of early/late apoptosis (2.07 ± 0.04%/7.65 ± 0.24%) and necrosis (1.39 ± 0.07%) owing to insufficient temperature to induce cell death. Eventually, the CRT expression in CT26 cells treated with 100 μg/ml of AuNR@SiO_2_-20 increased 2.22- to 2.51-fold and 1.73- to 1.8-fold compared to those treated with 50 or 200 μg/ml upon laser irradiation (Fig. 3G and Fig. S5). Furthermore, the extracellular release of ATP, HMGB1, and HSP70 from CT26 cells was 1.98- to 2.02-fold and 1.16- to 1.19-fold, 4.56- to 4.77-fold and 1.02- to 1.09-fold, and 2.46- to 2.6-fold and 2.2- to 2.31-fold higher after treatment with 100 μg/ml compared to 50 or 200 μg/ml of AuNR@SiO_2_-20 upon laser irradiation, respectively (Fig. 3H and Fig. S6). Taken together, these results demonstrate that AuNR@SiO_2_-20 treatment at a specific condition to induce mild PTT effectively leads to ICD in tumor cells, ultimately triggering a robust antitumor immune response.

### In vivo studies to induce AuNR@SiO_2_-20-mediated mild PTT within colon tumor tissues.

**Fig. 4.**
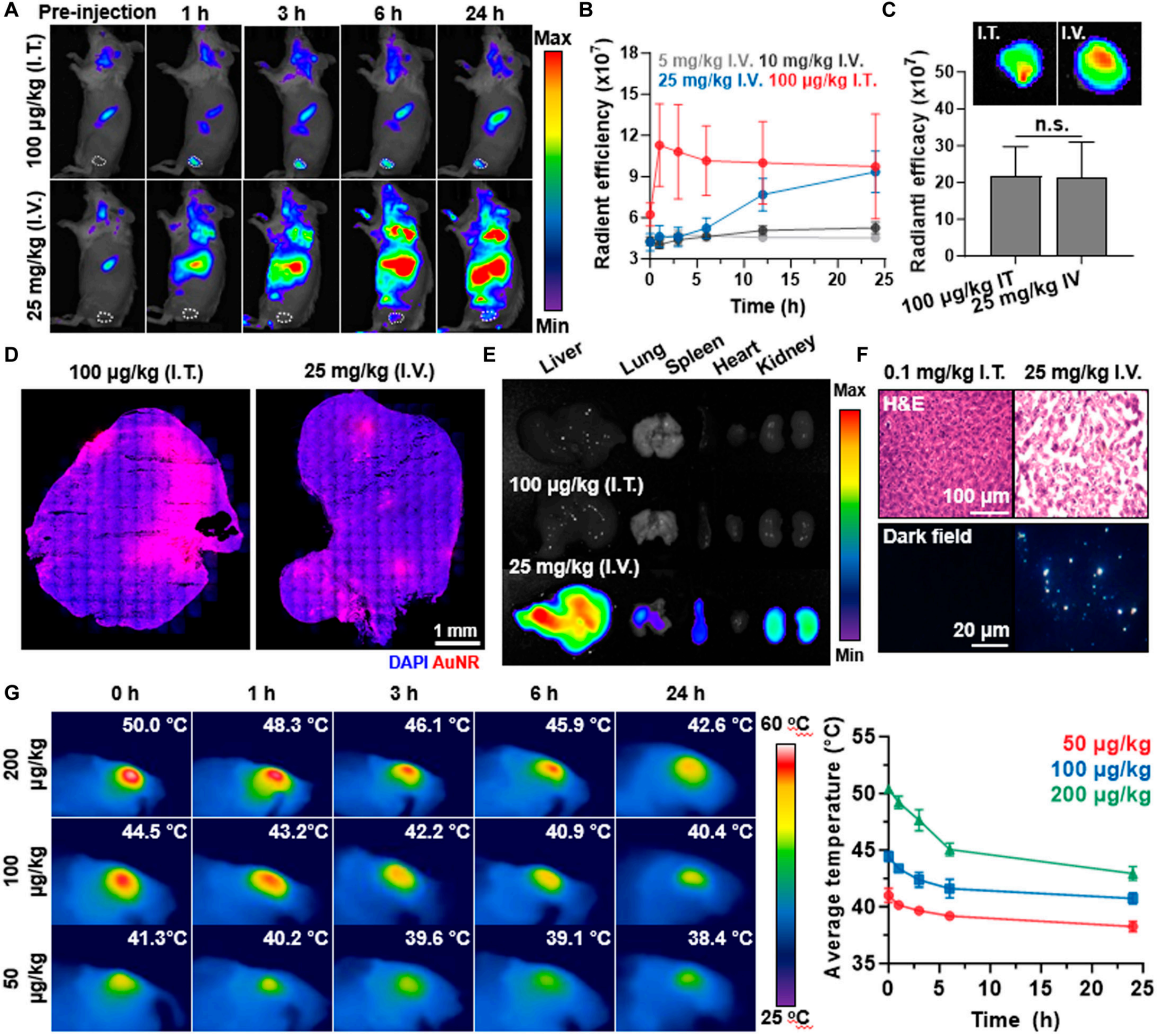
In vivo optimization studies to induce AuNR@SiO_2_-20-mediated mild PTT within colon tumor tissues. (A) NIRF images of CT26 tumor-bearing mice after 100 μg/kg I.T. or 25 mg/kg I.V. injection of AuNR@SiO_2_-20. (B) Quantitative analyses for the fluorescence intensities in tumor region (white dotted circle) of CT26 tumor-bearing mice after 100 μg/kg I.T. or 25 mg/kg I.V. injection of AuNR@SiO_2_-20. (C) NIRF images of tumor tissues from CT26 tumor-bearing mice after 24 h of 25 mg/kg I.V. administration or immediately after 100 μg/kg I.T. injection of AuNR@SiO_2_-20. (D) Fluorescence images of whole tumor areas after mice were treated with 24 h of 25 mg/kg I.V. administration or immediately after 100 μg/kg I.T. injection of AuNR@SiO_2_-20. (E) Ex vivo NIRF images of major organs from CT26 tumor-bearing mice after 24 h of 25 mg/kg I.V. administration or immediately after 100 μg/kg I.T. injection of AuNR@SiO_2_-20. (F) Dark field or H&E-stained liver tissues from CT26 tumor-bearing mice after 24 h of 25 mg/kg I.V. administration or immediately after 100 μg/kg I.T. injection of AuNR@SiO_2_-20. (G) In vivo photothermal images of CT26 tumor-bearing mice after 50, 100, or 200 μg/kg I.T. injection of AuNR@SiO_2_-20 upon laser irradiation for 4 min with a power of 1.0 W/cm^2^.

The administration route and treatment protocol details to efficiently induce mild PTT within tumor tissues were optimized in colon tumor-bearing mice, which are prepared by subcutaneous inoculation with 1 × 10^6^ CT26 cells into the left flank of BALB/c mice. The quantity of Cy5.5-AuNR@SiO_2_-20 in the tumor tissues was compared following administration with I.T. or I.V. routes. We postulated that I.T. injection can lead to the regression of primary tumors and metastatic tumors while minimizing in vivo toxicities, due to the potent antitumor immune response resulting from mild PTT through significant tumor accumulation with an excessively low material dosage compared to I.V. injection. Upon I.T. injection of 100 μg/kg Cy5.5-AuNR@SiO_2_-20 into CT26 tumor-bearing mice, their bright fluorescence was clearly observed within the tumor tissues (white dotted circle; Fig. 4A and B). To achieve a similar level of tumor accumulation to I.T. injected 100 μg/kg Cy5.5-AuNR@SiO_2_-20 through I.V. injection, a high dose of 25 mg/kg had to be administered, wherein the fluorescence intensity within the tumor tissues was nearly comparable at 24 h after I.V. administration of 25 mg/kg Cy5.5-AuNR@SiO_2_-20 to that immediately after 100 μg/kg of I.T. injection (Fig. 4C). However, I.V. administration with 0.1, 5, or 10 mg/kg of Cy5.5-AuNR@SiO_2_-20 was insufficient to achieve comparable tumor accumulation when compared to I.T. injection with 100 μg/kg; as a control, the saline-treated CT-26 tumor-bearing mice exhibited very weak autofluorescence throughout the whole body (Fig. S7). In addition, Cy5.5-AuNR@SiO_2_-20 remain localized at the administration site after subcutaneous (S.C.) and I.P. injection (25 mg/kg), showing limited tumor accumulation compared to I.T. route (Fig. S8). Histological analyses were also performed to further assess the difference in the distribution of Cy5.5-AuNR@SiO_2_-20 within the tumor tissues after 100 μg/kg I.T. or 25 mg/kg I.V. injection (Fig. 4D and Fig. S9). Interestingly, Cy5.5-AuNR@SiO_2_-20 (red color) remained confined to the injection site within the tumor tissues immediately after I.T. injection, whereas that was evenly distributed throughout the entire tumor area after 24 h of I.V. administration. Based on these results, we hypothesized that the I.T. route is suitable and effective for promoting mild PTT efficiently and precisely within tumor tissues through AuNR@SiO_2_-20, which accumulates significantly at the injection site. Hence, significant antitumor efficacy could also be anticipated even in the remaining tumor margin where AuNR@SiO_2_-20 does not exist as well as rechallenged tumors, via abscopal effect by a potent antitumor immune response resulting from mild PTT [34]. Notably, the high dose of 25 mg/kg Cy5.5-AuNR@SiO_2_-20 led to significant accumulation in off-target tissues, including the liver, lung, spleen, and kidney, eventually causing severe structural abnormalities due to its severe toxicity 24 h after I.V. administration (Fig. 4E and F and Fig. S10). Furthermore, a remarkably reduced risk of potential toxicity in off-target tissues can also be expected due to absolutely low material dosage through I.T. administration.

Next, a detailed treatment protocol for AuNR@SiO_2_-20 to induce mild PTT within tumor tissues was investigated in CT26 tumor-bearing mice. Different AuNR@SiO_2_-20 doses of 50, 100, or 200 μg/kg were I.T. injected into the mice, followed by photothermal imaging at the indicated time points after the tumor tissues were locally irradiated for varying durations with a power of 1.0 W/cm^2^ (Fig. 4G). While such irradiation is a very strong condition causing tissue burning, we aimed to induce mild temperature with minimal administration of AuNR@SiO_2_-20, which exhibits superior photothermal effects, through IT injection of less than 10% compared to previous studies [35]. Local treatment with 100 μg/kg AuNR@SiO_2_-20 upon laser irradiation for 4 min precisely promoted a mild temperature of 44.5 °C within the tumor tissues. In the case of mice treated with 50 or 200 μg/kg AuNR@SiO_2_-20 and laser irradiation, the local temperature within the tumor tissues was increased up to 41.3 and 50 °C in the same laser irradiation condition, respectively. Notably, the treatment with 50 or 200 μg/kg AuNR@SiO_2_-20 resulted in temperatures either lower or higher than the mild temperature in tumor tissues under all laser irradiation conditions ranging from 1 to 6 min with a power of 1.0 W/cm^2^. These results allowed for the optimization for AuNR@SiO_2_-20-mediated mild PTT in vivo, which can precisely and reproducibly induce mild temperatures within tumor tissues without any potential nonspecific toxicities.

### Antitumor efficacy and immune responses by AuNR@SiO_2_-mediated mild PTT in colon tumor models.

**Fig. 5.**
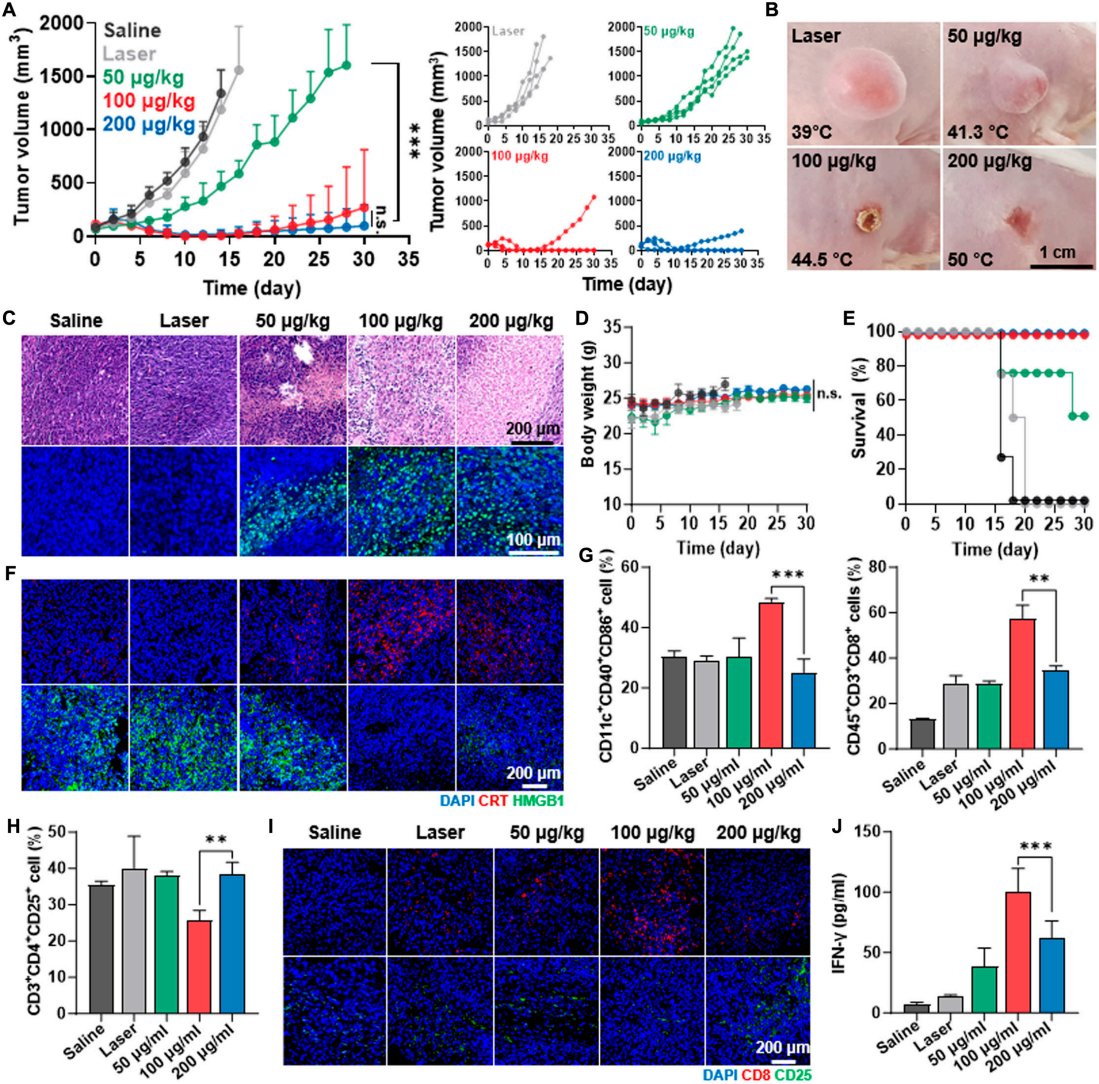
Antitumor efficacy and immune responses by AuNR@SiO_2_-mediated mild PTT in colon tumor models. (A) Tumor growth curves of CT26 tumor-bearing mice after 50, 100, or 200 μg/kg via I.T. injection of AuNR@SiO_2_-20 upon laser irradiation for 4 min with a power of 1.0 W/cm^2^. (B) Optical images of tumor tissues of mice on day 16 after 50, 100, or 200 μg/kg I.T. injection of AuNR@SiO_2_-20 upon laser irradiation for 4 min with a power of 1.0 W/cm^2^. Mice in the laser group treated only with laser irradiation in a same protocol. (C) Tumor tissues stained with H&E or TUNEL on day 16 after treatment. (D and E) Body weight changes and survival of mice during treatment. (F) Tumor tissues stained with anti-CRT or anti-HMGB1 antibodies on day 16 after treatment. (G and H) Population of mature DCs (CD11c^+^CD40^+^CD86^+^), CTLs (CD45^+^CD3^+^CD8^+^), and T_regs_ (CD3^+^CD4^+^CD25^+^) within the tumor tissues on day 16 after treatment. (I) Tumor tissues stained with anti-CD8 or anti-CD25 antibodies on day 16 after treatment. (J) Serum levels of IFN-γ on day 16 after treatment.

The comparative studies were conducted with AuNR@SiO_2_-20 to confirm whether promoting a mild temperature (44.5 °C) within the tumor tissues leads to an enhanced antitumor immune response compared to lower (41.3 °C) and higher (50 °C) temperatures. For these analyses, CT26 tumor-bearing mice were randomly divided into five groups: (a) saline, (b) laser, (c) 50 μg/kg AuNR@SiO_2_-20 with laser (+L), (d) 100 μg/kg AuNR@SiO_2_-20 (+L), and (e) 200 μg/kg AuNR@SiO_2_-20 (+L). Each dose of AuNR@SiO_2_-20 was I.T. injected into the mice, followed by laser irradiation of the tumor tissues with a power of 1.0 W/cm^2^ for 4 min. The mice in the 100 (20.33 ± 40.67 mm^3^) or 200 (29.79 ± 59.58 mm^3^) μg/kg AuNR@SiO_2_-20 (+L) groups exhibited significantly delayed tumor growth on day 16 compared to those in the saline (2,082.72 ± 539.96 mm^3^), laser (1,559.9 ± 489.87 mm^3^), and 50 μg/kg AuNR@SiO_2_-20 (+L; 587.96 ± 122.16 mm^3^) groups, respectively (Fig. 5A and Fig. S11). The photographs of tumor tissues on day 16 after treatment also showed outstanding antitumor efficacy in the 100 or 200 μg/kg AuNR@SiO_2_-20 (+L) groups (Fig. 5B). Histological analyses of tumor tissues stained with hematoxylin and eosin (H&E) or TUNEL confirmed significantly up-regulated structural abnormalities and apoptosis after treatment with 100 or 200 μg/kg AuNR@SiO_2_-20 (+L) compared to other treatments (Fig. 5C). Extensive apoptotic cell death within tumor tissues was also demonstrated, as the 100 μg/kg AuNR@SiO_2_-20 (+L) group showed elevated caspase-3 expression compared to the other groups (Fig. S12). During the treatments, no significant body weight loss was observed in mice from all the groups (Fig. 5D). The median survival of mice treated with saline, laser, or 50 μg/kg AuNR@SiO_2_-20 (+L) was determined to be 16, 18, or 28 days owing to tumor growth, respectively, while no deaths occurred in the 100 or 200 μg/kg AuNR@SiO_2_-20 (+L) groups (Fig. 5E). These results indicate that either mild or higher temperatures within the tumor tissues induced by 100 or 200 μg/kg AuNR@SiO_2_-20 (+L) resulted in a similar antitumor efficacy in eradicating the primary tumors.

However, the antitumor immune responses within the tumor microenvironment exhibited significant up-regulation when a mild temperature was induced in the tumors using 100 μg/kg AuNR@SiO_2_-20 (+L) compared to the higher temperature by a dosage of 200 μg/kg AuNR@SiO_2_-20 (+L). First, the highest levels of CRT expression (red color) were clearly observed within the tumor tissues in the 100 μg/kg AuNR@SiO_2_-20 (+L) group compared to 200 μg/kg AuNR@SiO_2_-20 (+L) and all other groups on day 16 (Fig. 5F). Tumor tissues in the 100 μg/kg AuNR@SiO_2_-20 (+L) group also exhibited only a small amount of HMGB1 fluorescence signals (green color) due to their extensive extracellular release, a result of potent ICD in the tumor cells. The relatively low levels of DAMPs in tumor tissues treated with 200 μg/kg AuNR@SiO_2_-20 (+L) can be attributed to severe inflammatory responses resulting from necrotic cell death owing to an excessively higher temperature. In addition, the population of mature DCs (CD11c^+^CD40^+^CD86^+^), CTLs (CD45^+^CD3^+^CD8^+^), and regulatory T lymphocytes (T_regs_; CD3^+^CD4^+^CD25^+^) in the tumor tissues was measured by flow cytometry on day 16 after treatment. As expected, the 100 μg/kg AuNR@SiO_2_-20 (+L) group revealed a significantly higher population of DCs and CTLs in tumor tissues than the 200 μg/kg AuNR@SiO_2_-20 (+L; 1.89- to 1.96-fold DCs and 1.52- to 1.63-fold CTLs), 50 μg/kg AuNR@SiO_2_-20 (+L; 1.66- to 1.7-fold and 1.91- to 1.99-fold), laser (1.7- to 1.76-fold and 1.88- to 1.99-fold), and saline (1.58- to 1.68-fold and 4.32- to 4.49-fold) groups, respectively (Fig. 5G). In contrast, the population of T_regs_ within the tumor tissues was greatly down-regulated in the 100 μg/kg AuNR@SiO_2_-20 (+L) group, resulting in a significant increase in the ratio of CTLs to T_regs_ compared to other groups (Fig. 5H). This significant increase in CTLs (red color) along with the decrease in T_regs_ (green color) within the tumors by mild temperature after treatment with 100 μg/kg AuNR@SiO_2_-20 (+L) was further confirmed via histological analyses (Fig. 5I). Finally, a high quantity of interferon-γ (IFN-γ) was observed in the tumor supernatants from the 100 μg/kg AuNR@SiO_2_-20 (+L) group owing to the elevated IFN-γ release from activated T cells into the tumor microenvironment (Fig. 5J). Taken together, these results demonstrate that treatment with 100 μg/kg AuNR@SiO_2_-20 (+L) effectively reverses the ITM into an immune-responsive milieu that is expected a favorable response against ICB therapy, by inducing a mild temperature in the tumor tissues.

### Antitumor immune response by the combination of AuNR@SiO_2_-20-mediated mild PTT with ICB therapy

**Fig. 6.**
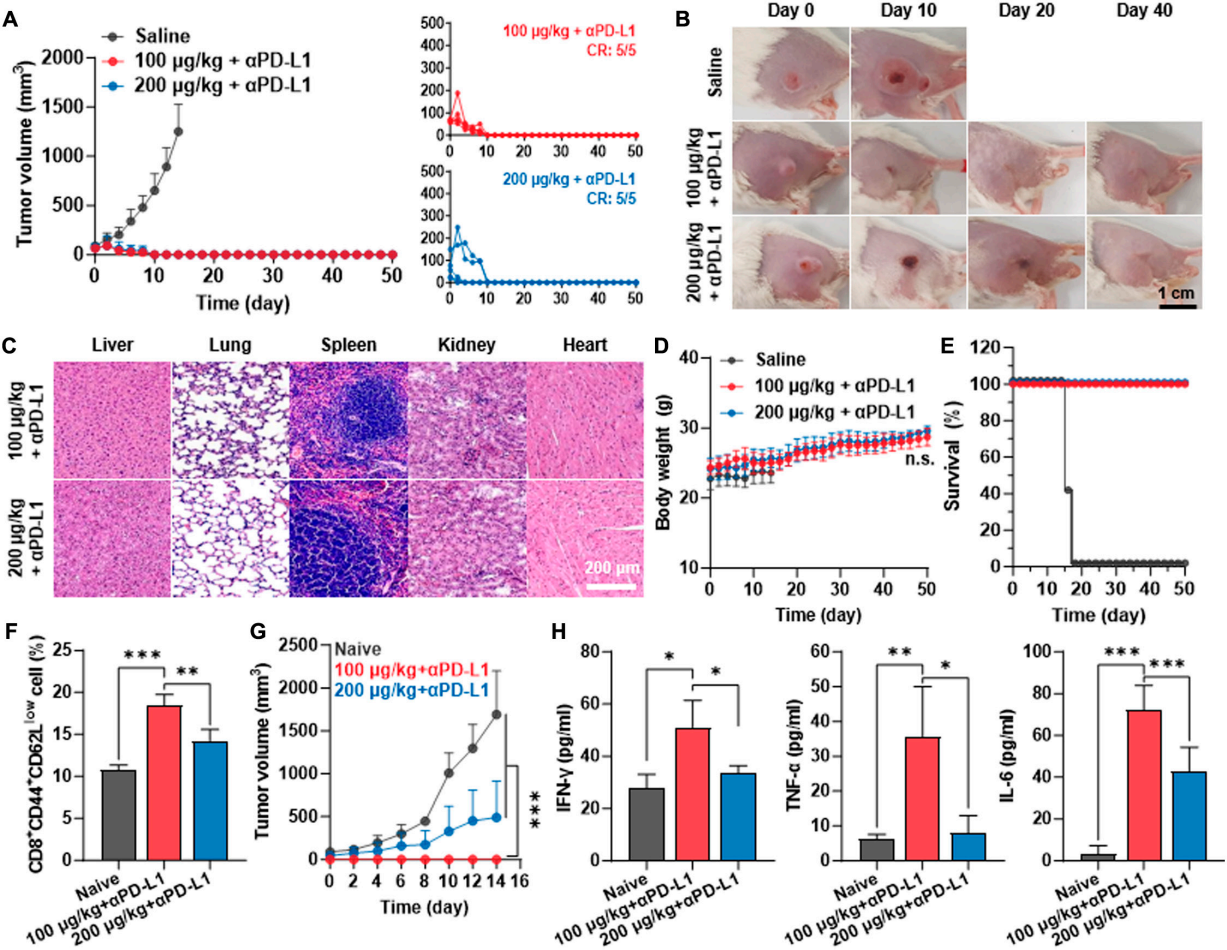
Antitumor immune response by combination of AuNR@SiO_2_-20-mediated mild PTT with ICB therapy. (A) Tumor growth curves of CT26 tumor-bearing mice in saline, 100 μg/kg AuNR@SiO_2_-20 plus αPD-L1 (+L), and 200 μg/kg AuNR@SiO_2_-20 plus αPD-L1 (+L) groups. (B) Optical images of tumor tissues of mice in saline, 100 μg/kg AuNR@SiO_2_-20 plus αPD-L1 (+L), and 200 μg/kg AuNR@SiO_2_-20 plus αPD-L1 (+L) groups. (C) Tumor tissues stained with H&E on day 12 after treatment. (D and E) Body weight changes and survival of mice during treatment. (F) Population of splenic effector/memory T cells (T_em_; CD8^+^CD44^+^CD62L^low^) on day 12 after treatment. (G) Rechallenged CT26 tumor growth in CR mice that experienced a complete tumor regression on day 12 after treatment with 100 μg/kg AuNR@SiO_2_-20 plus αPD-L1 (+L) or 200 μg/kg AuNR@SiO_2_-20 plus αPD-L1 (+L). (H) Serum levels of IFN-γ, TNF-α, and IL-6 on day 16 after treatment.

With the optimal conditions to promote AuNR@SiO_2_-20-mediated mild PTT in vivo, the antitumor immune responses by its combinatorial treatment with anti-PD-L1 antibody (αPD-L1) were assessed in colon tumor models. More importantly, we intended to evaluate whether PTT with mild temperature enhances the ICB therapy efficiency in comparison to that with excessively higher temperature via their significant effects in reversing ITM into an immune-responsive milieu. Hence, the mice were randomly divided into three groups: (a) saline, (b) 100 μg/kg AuNR@SiO_2_-20 plus αPD-L1 (+L), and (c) 200 μg/kg AuNR@SiO_2_-20 plus αPD-L1 (+L). The AuNR@SiO_2_-20 and laser irradiation were treated with the same protocol as described in Fig. 5, and 10 mg/kg αPD-L1 was administered through I.P. injection. As expected, treatment with either 100 or 200 μg/kg AuNR@SiO_2_-20 plus αPD-L1 (+L) resulted in significant tumor inhibition compared to the saline group, but there were no significant differences in antitumor efficacy to eradicate the primary tumors between both groups (Fig. 6A and Fig. S13). Furthermore, mice in both 100 or 200 μg/kg AuNR@SiO_2_-20 plus αPD-L1 (+L) groups experienced 100% complete tumor regression (CR: 5/5) within 12 days of treatment (Fig. 6B). During treatments, no significant body weight loss or nonspecific toxicity to the major organs, including the liver, lung, spleen, kidney, and heart, was observed in all the groups (Fig. 6C and Fig. S14). Eventually, the median survival of the saline group was determined to be 16 days after treatment owing to tumor progression, whereas mice in the 100 or 200 μg/kg AuNR@SiO_2_-20 plus αPD-L1 (+L) groups survived for over 50 days (Fig. 6D). Next, the effects of treatment with 100 or 200 μg/kg AuNR@SiO_2_-20 plus αPD-L1 (+L) on establishing in vivo immunological memory, which enables the host’s immune system to prevent recurrence by previously encountered tumor cells, were compared in mice that experienced a CR (CR mice). The population of effector/memory T cells (T_em_), a hallmark of immunological memory following cancer immunization, was significantly higher in CR mice from 100 μg/kg AuNR@SiO_2_-20 plus αPD-L1 (+L; 18.43 ± 1.31%) group compared to those treated with 200 μg/kg AuNR@SiO_2_-20 plus αPD-L1 (+L; 14.23 ± 1.36%; Fig. 6E). In addition, CR mice in both groups were also rechallenged with 1 × 10^6^ CT26 tumor cells on day 50 after initial treatments. Consequently, CR mice in the 100 μg/kg AuNR@SiO_2_-20 plus αPD-L1 (+L) group exhibited a remarkable resistance to rechallenged tumor growth for 14 days, in contrast to rapid growth observed in naive mice (Fig. 6F and Fig. S15). While the effects to inhibit the rechallenged tumor growth were also observed in CR mice from 200 μg/kg AuNR@SiO_2_-20 plus αPD-L1 (+L) group, those were significantly tenuous than in the 100 μg/kg AuNR@SiO_2_-20 plus αPD-L1 (+L) group. This enhanced response is attributed to the robust immunological memory of the immune system to eradicate the rechallenged tumors more rapidly and effectively in vivo after treatment with 100 μg/kg AuNR@SiO_2_-20 plus αPD-L1 (+L). Subsequently, the serum levels of cytokines, including IFN-γ, tumor necrosis factor-α (TNF-α), and interleukin-6 (IL-6), were significantly increased in CR mice treated with 100 μg/kg AuNR@SiO_2_-20 plus αPD-L1 (+L), as compared to those observed in naive mice or CR mice treated with 200 μg/kg AuNR@SiO_2_-20 plus αPD-L1 (+L), following tumor rechallenge (Fig. 6G). Based on these findings, this study demonstrates that the combination of 100 μg/kg AuNR@SiO_2_-20 (+L) with αPD-L1 achieves 100% complete regression of primary tumors while significantly minimizing side effects during ICB therapy due to the absolutely reduced material dosage via I.T. administration. In addition, this approach also establishes robust and durable immunological memory in vivo, effectively preventing tumor recurrence.

## Conclusion

The current ICB therapy has faced a formidable challenge due to the low response rates of patients with ITM, characterized by low tumor-associated antigens, T lymphocytes, and high immune regulatory cells. As ICD is increasingly recognized as a crucial strategy for enhancing cancer immunogenicity, diverse approaches have been explored to reverse ITM into an immune-responsive milieu and ultimately increase the ICB therapy efficiency. Recently, PTT at mild temperatures ranging from 44 to 45 °C has received considerable attention due to its remarkable ability to promote potent ICD within tumor cells, consequently triggering antitumor immune responses. This study provided extensive optimization studies involving material design and preparation, administration routes, and treatment details to precisely and reproducibly induce mild temperature within tumor tissues. The most significant discovery is that the tumor cells treated with AuNR@SiO_2_ under mild temperature elicit an outstanding antitumor immune response compared to those exposed to lower or higher temperatures, following laser irradiation under cultured conditions. A significant effect of AuNR@SiO_2_-20 in inducing mild temperatures within tumor tissues was also observed in colon tumor models, leading to an increase in TILs as a result of remarkable ICD accompanied by DAMPs. Ultimately, the combination of AuNR@SiO_2_-20 and anti-PD-L1 therapy led to 100% CR of primary tumors and also effectively prevented tumor recurrence through enhanced antitumor efficacy and immune responses. Meanwhile, the potential risk of side effects from the administration of inorganic materials can also be significantly reduced due to the absolutely low dosages via I.T. administration. In summary, this study suggests that the development of a robust methodology capable of continuously inducing mild temperature within the ITM holds promise as an approach to potentiate the ICB therapies and ultimately eradicate the tumors.

## Data Availability

All relevant data are available within the article and its supplementary information files, or available from the corresponding authors upon reasonable requests.
