# Supplementary material for: Accurately Controlled Tumor Temperature with Silica-Coated Gold Nanorods for Optimal Immune Checkpoint Blockade Therapy
Source: Biomater Res. 2024 Apr 29;28:0024. doi: 10.34133/bmr.0024 (PMC11062504; doi:10.34133/bmr.0024)
Supplement: Supplementary 1 — Figs. S1 to S15 [file bmr.0024.f1.docx]

**Supplementary Information for**

Accurately controlled tumor temperature with silica-coated gold nanorods for optimal immune checkpoint blockade therapy

Wan Su Yun^1,2,#^, Wonseok Yang^2,#^, Man Kyu Shim^3,#^, Sukyung Song^1^, Jiwoong Choi^3^, Jeongrae Kim^1,2^, Jinseong Kim^1^, Yujeong Moon^3^, SeongHoon Jo^3^, Dong-Kwon Lim^2,4,5,*^, Kwangmeyung Kim^1,*^

^1^ College of Pharmacy, Graduate School of Pharmaceutical Sciences, Ewha Womans University, Seoul 03760, Republic of Korea

^2^ KU-KIST Graduate School of Converging Science and Technology, Korea University, 145 Anam-ro, Seongbuk-gu, Seoul, 02841, Republic of Korea

^3^ Medicinal Materials Research Center, Biomedical Research Division, Korea Institute of Science and Technology (KIST), Seoul, 02792, Republic of Korea

^4^ Department of Integrative Energy Engineering, Korea University, 145 Anam-ro, Seongbuk-gu,
Seoul 02841, Republic of Korea

^5^ Brain Science Institute, Korea Institute of Science and Technology (KIST), 5, Hwarang-ro 14-gil, Seongbuk-gu, Seoul 02792, Republic of Korea

^#^These authors contributed equally to this work.

*Correspondence and requests for materials should be addressed to **K. Kim** (E-mail: kimkm@ewha.ac.kr) or **D.-K. Lim** (E-mail: dklim@korea.ac.kr).

**Keywords:** Gold nanorod, cancer immunotherapy; immune checkpoint blockade; photothermal therapy; mild temperature; immunogenic cell death

**
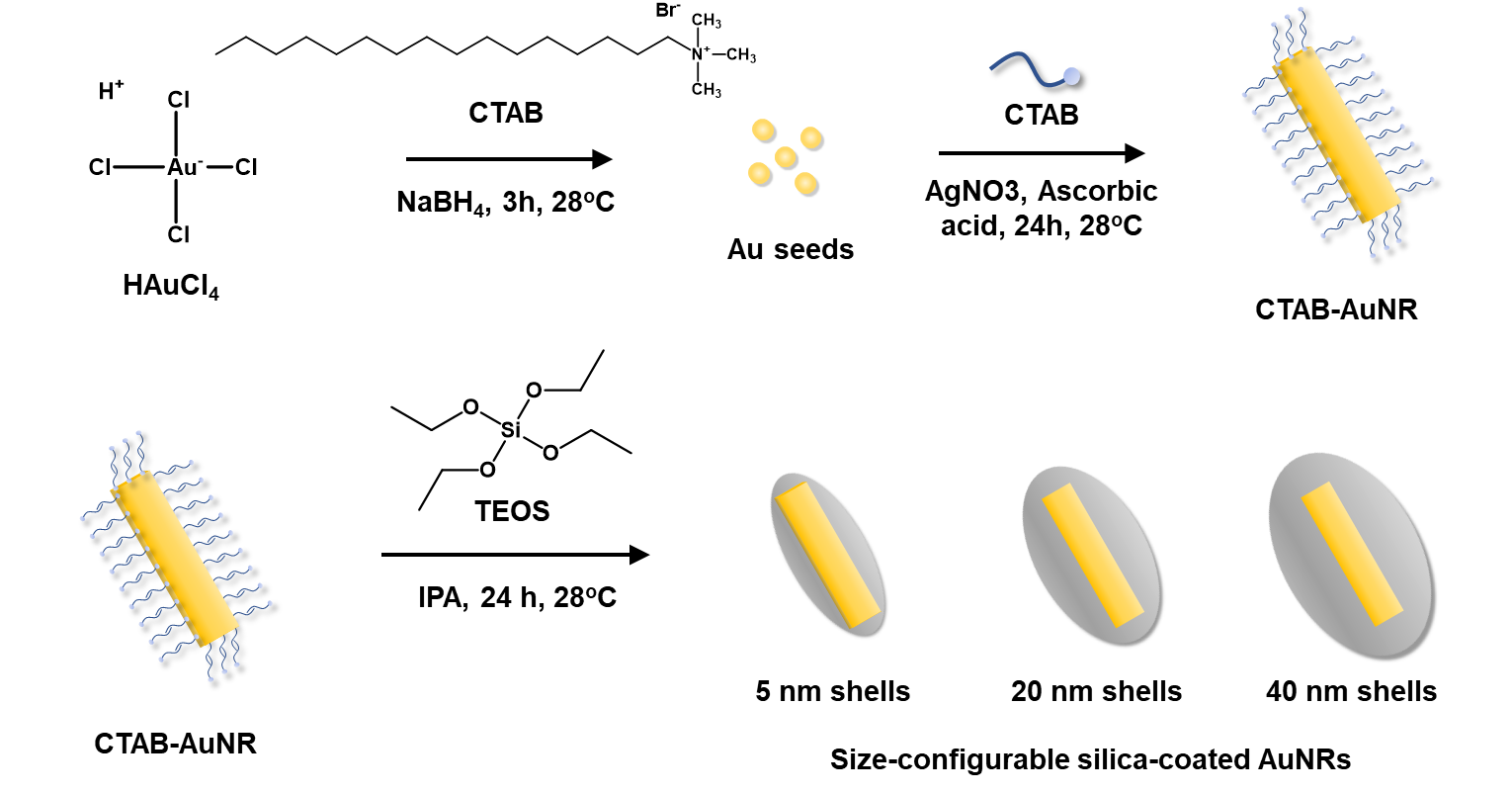
**

**Figure S1.** Synthetic scheme to prepare AuNR@SiO_2_ with varying silica shell thickness. At first, gold seeds were prepared by reduction of gold (III) chloride trihydrate. Then, CTAB-AuNRs were synthesized through a seed-mediated growth method. Second CTAB bilayers were reacted with varying quantities of TEOS for target silica shell thickness.


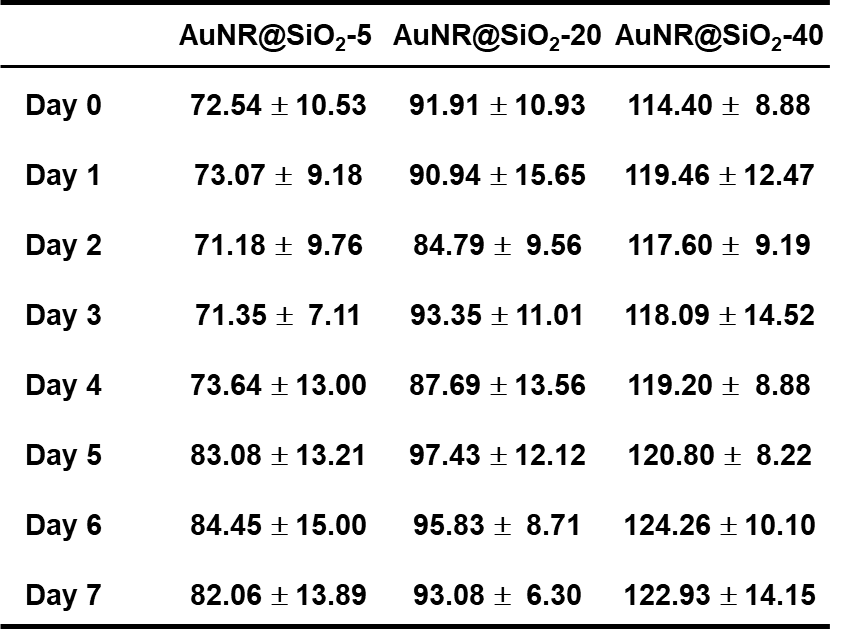


**Figure S2.** Detail information on hydrodynamic size of AuNR@SiO_2_-5, AuNR@SiO_2_-5, and AuNR@SiO_2_-20 upon incubation in saline for 7 days.


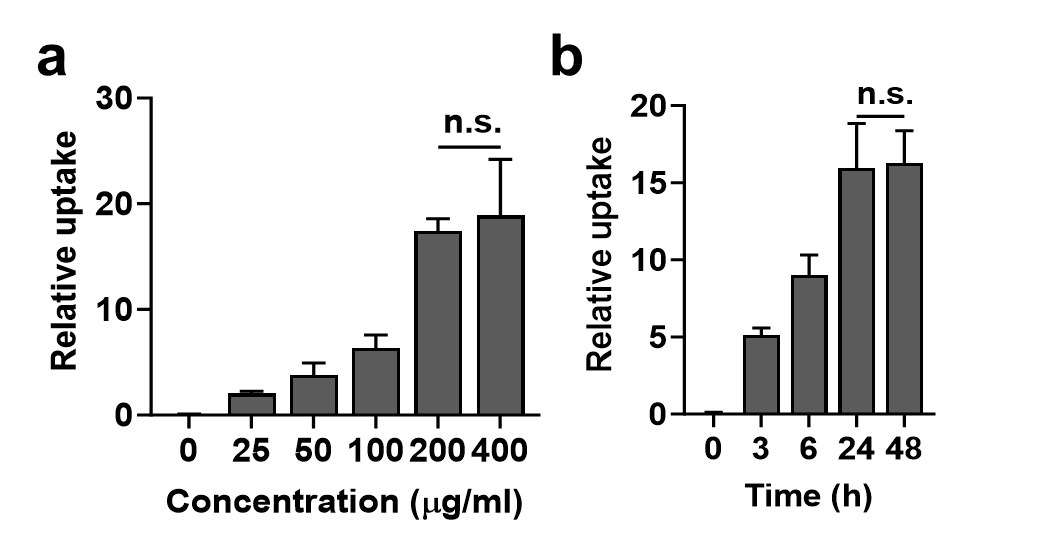


**Figure S3.** **(a)** Quantitative analyses of the quantity of AuNR@SiO_2_-20 in CT26 cells after treatment with different concentrations ranging from 0 to 400 μg/ml for 48 h. **(b)** Quantitative analyses of the quantity of AuNR@SiO_2_-20 in CT26 cells after 200 μg/ml treatment for varying incubation time.

**Figure S4.** The temperature within CT26 cells treated with 50, 100, or 200 μg/ml upon laser irradiation.


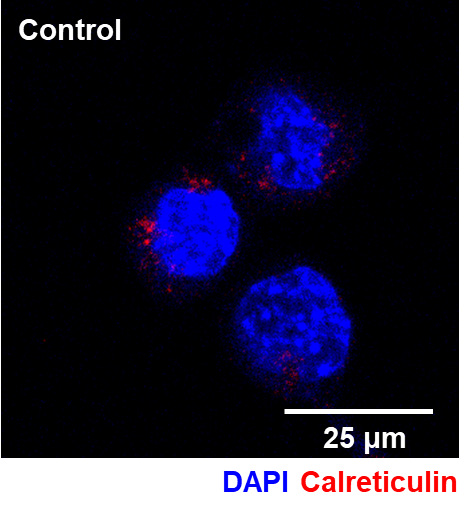


**Figure S5.** The expression of CRT in non-treated CT26 cells.


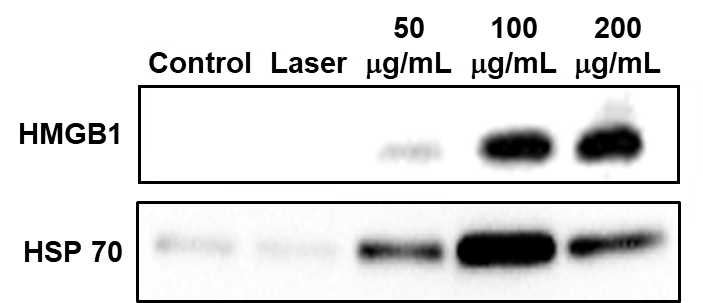


**Figure S6.** Western blot analysis to assess the extracellular release of HMGB1, and HSP70 from CT26 cells after 24 h of treatment with 50, 100, or 200 μg/ml AuNR@SiO_2_-20 upon laser irradiation for 3 min with power of 1.0 W/cm^2^.

**
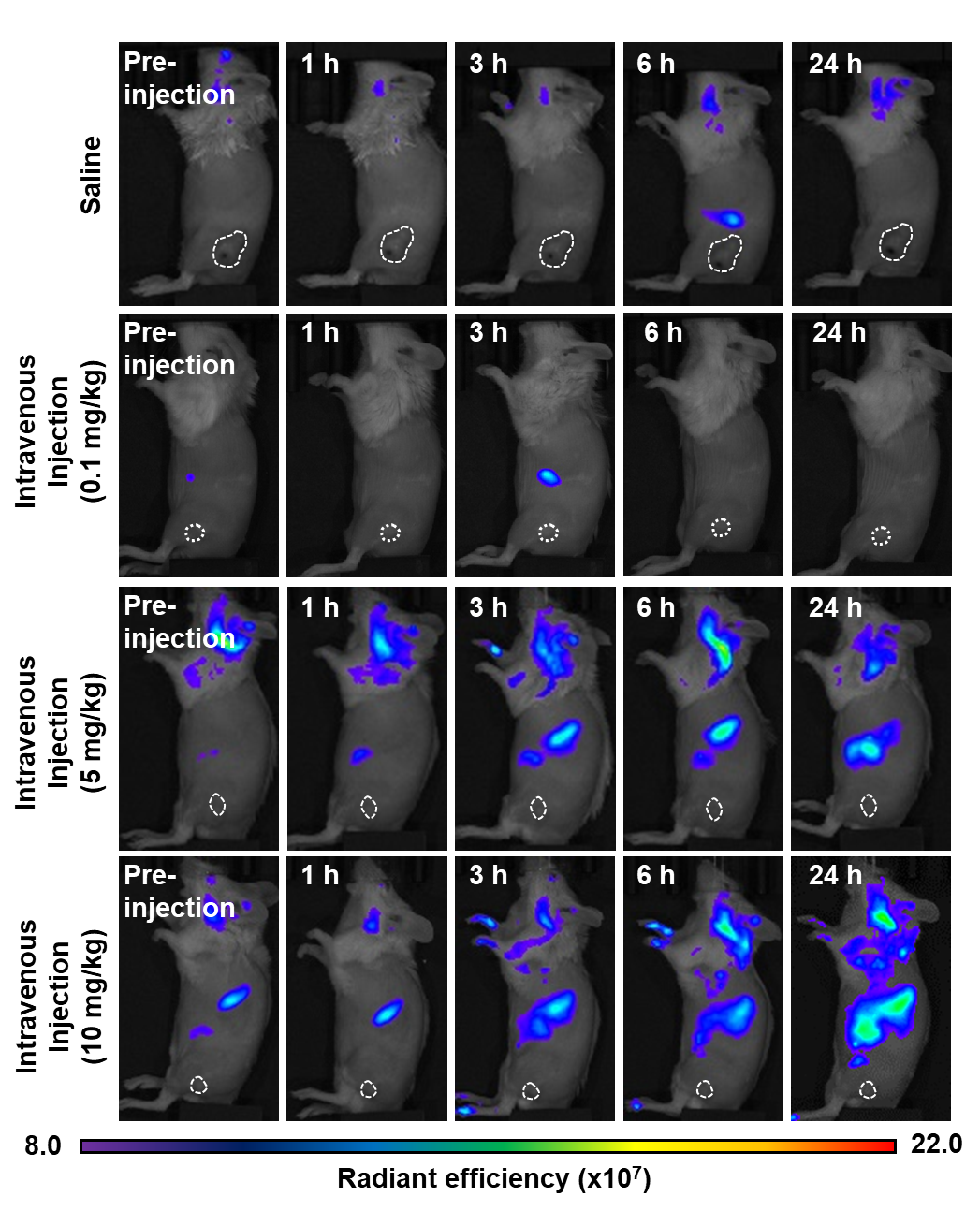
**

**Figure S7.** NIRF images of CT26 tumor-bearing mice after 0.1, 5 or 10 mg/kg I.V. injection of AuNR@SiO_2_-20.


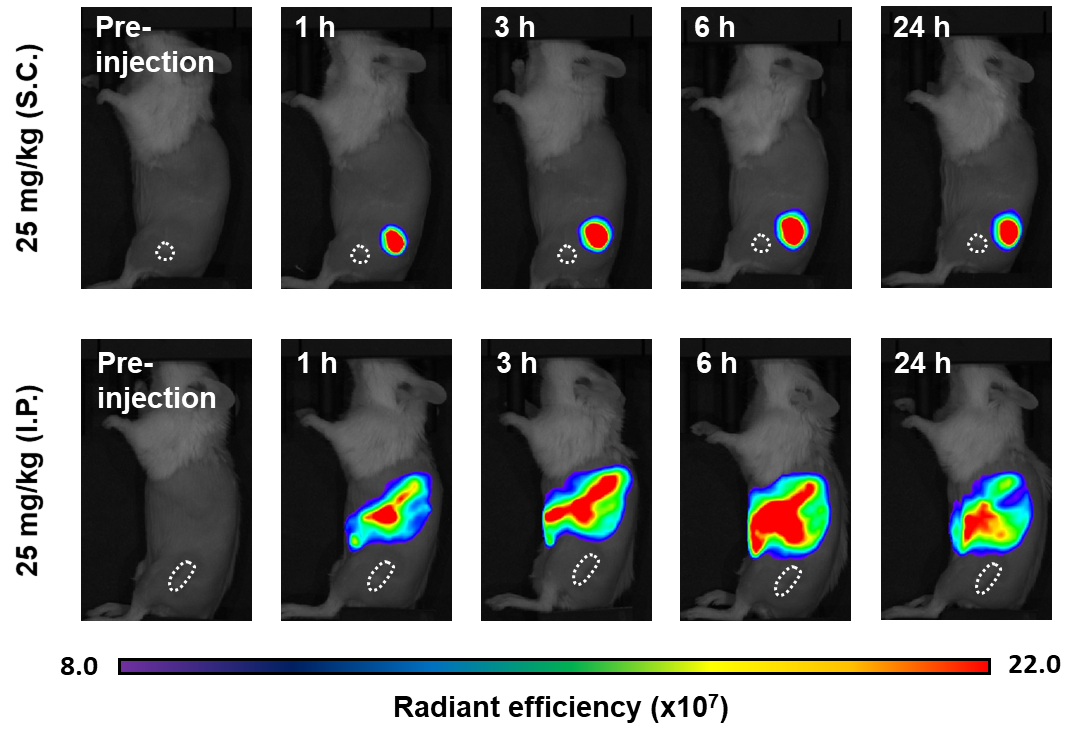


**Figure S8.** NIRF images of CT26 tumor-bearing mice after 25 mg/kg of S.C or I.P. injection of AuNR@SiO_2_-20.


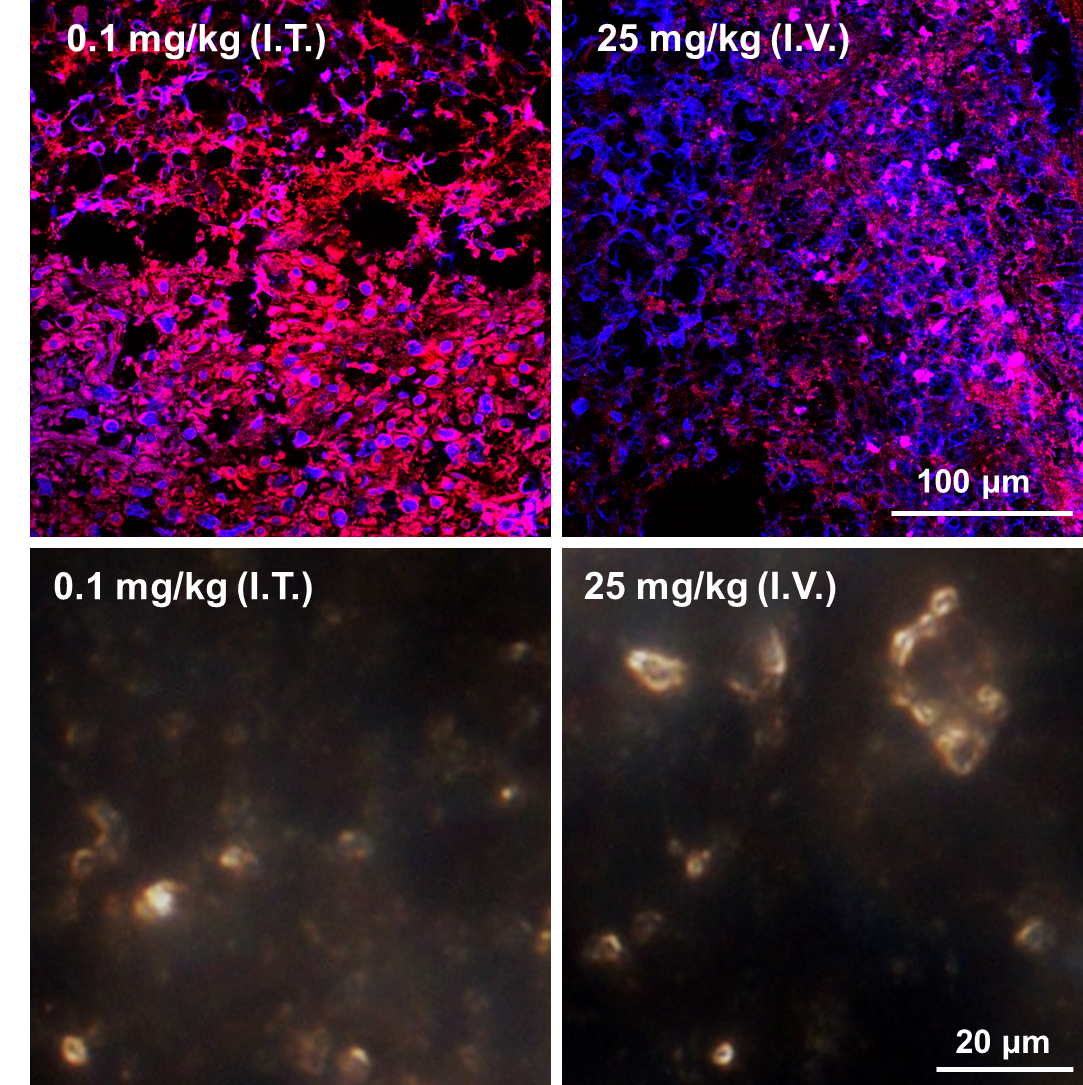


**Figure S9.** NIRF or dark field images of tumor tissues from CT26 tumor-bearing mice after 24 h of 25 mg/kg I.V. administration or immediately after 100 μg/kg I.T. injection of AuNR@SiO_2_-20.


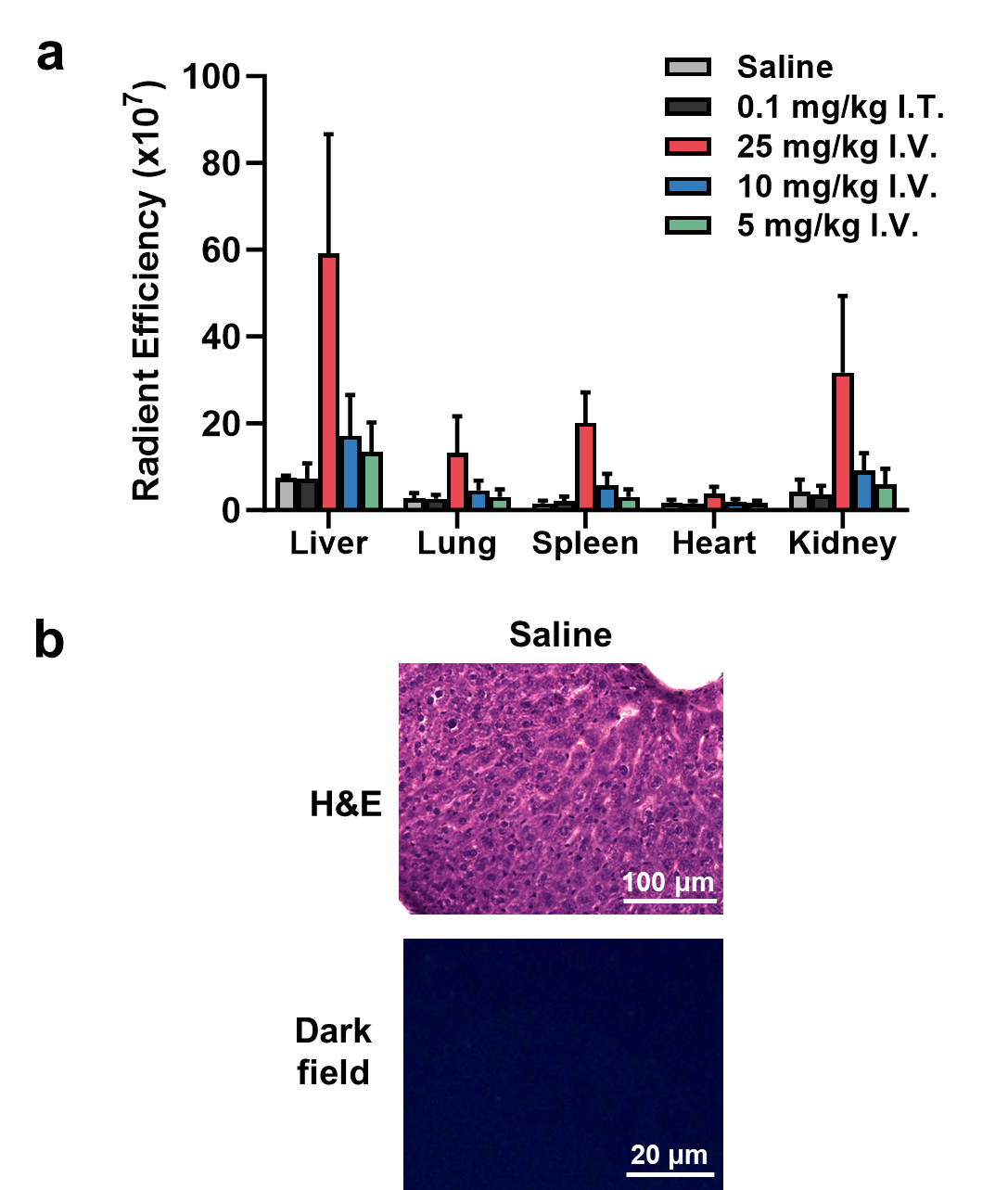


**Figure S10.** **(a)** Quantitative analysis of fluorescence intensities of AuNR@SiO_2_-20 in major organs after 24 h of 5, 10, or 25 mg/kg intravenous injection, or immediately after 0.1 mg/kg intratumoral injection. **(b)** Dark field or H&E-stained liver tissues from CT26 tumor-bearing mice in the Saline group.


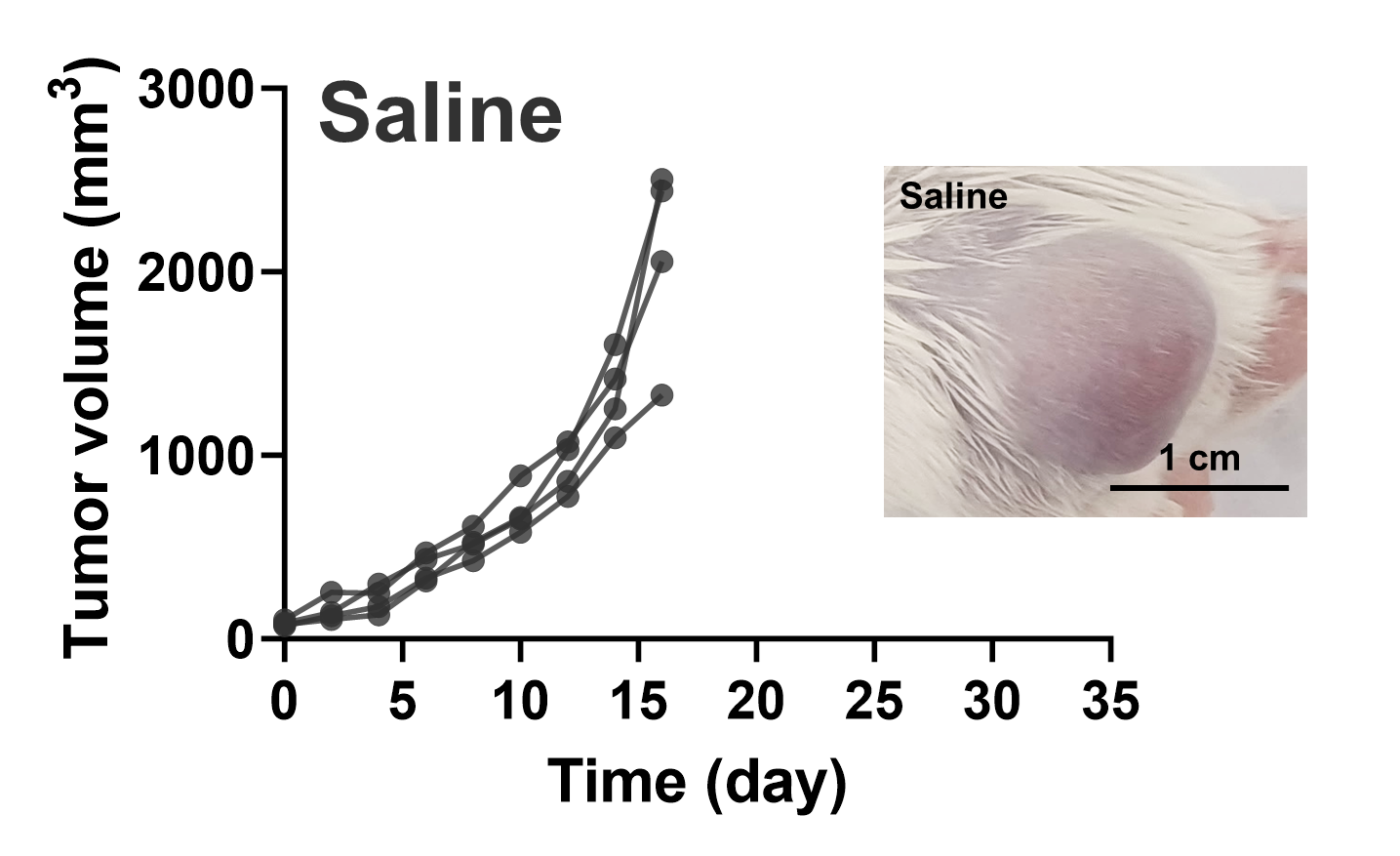


**Figure S11.** Tumor growth and optical images of tumors on day 16 of mice in saline group.


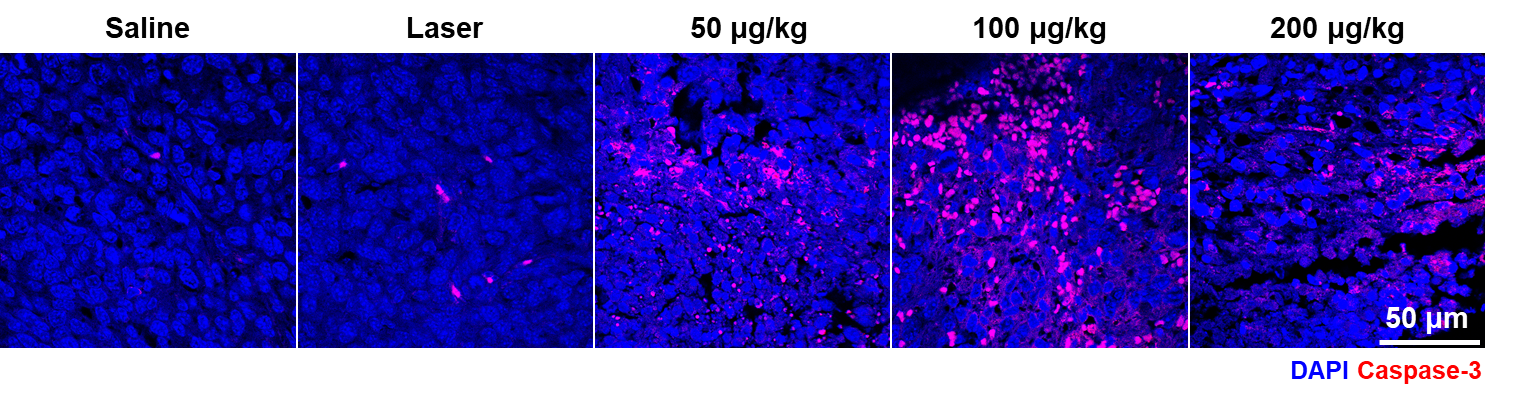


**Figure S12**. Tumor tissues stained with anti-caspase-3 antibody on day 16 after treatment.

**Figure S13.** Tumor growth curves of CT26 tumor-bearing mice in Saline group,


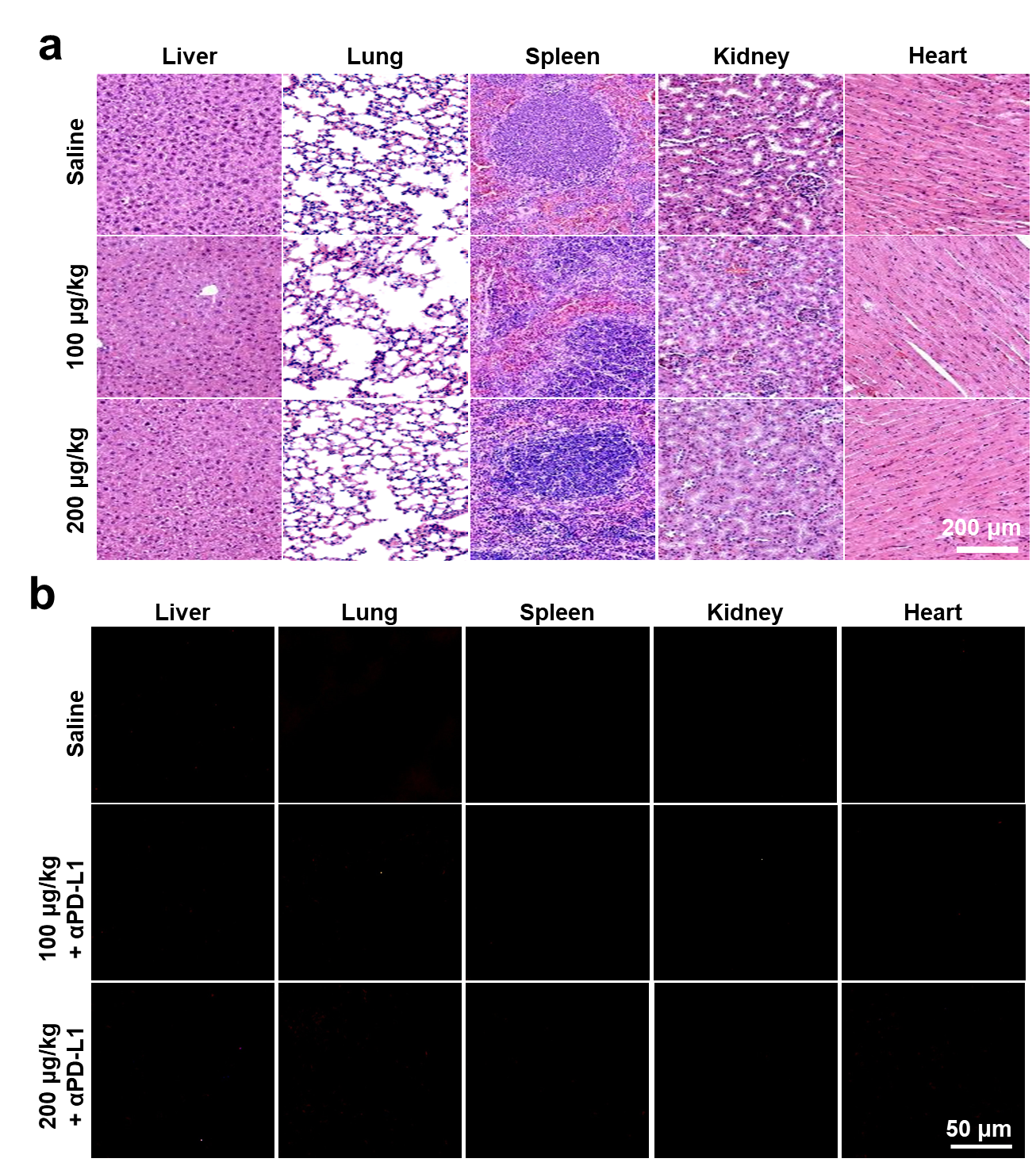


**Figure S14.** **(a)** Major organs stained with H&E on day 12 in saline, 100 or 200 μg/kg AuNR@SiO_2_-20 groups. **(b)** Dark field images of major organs of mice in Saline, 100 μg/kg AuNR@SiO_2_-20 plus αPD-L1 (+L), and 200 μg/kg AuNR@SiO_2_-20 plus αPD-L1 (+L) groups.


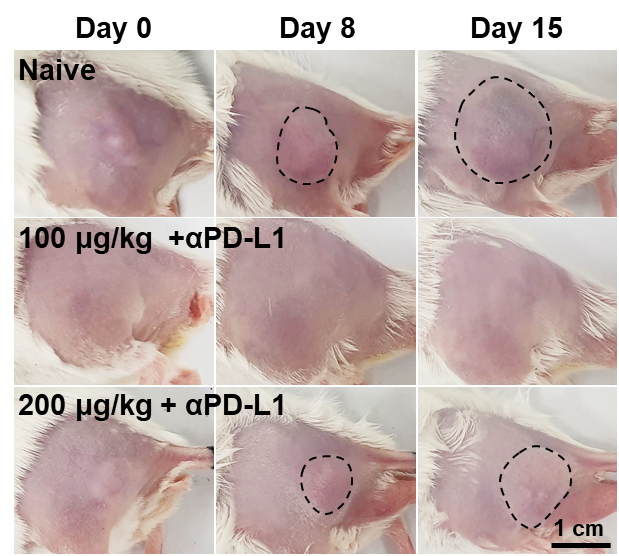


**Figure S15.** Optical images of rechallenged tumors of mice in Saline, 100 μg/kg AuNR@SiO_2_-20 plus αPD-L1 (+L), and 200 μg/kg AuNR@SiO_2_-20 plus αPD-L1 (+L) groups.
